# Key Mechanistic Features in Palladium-Catalyzed Methylcyclopropanation of Norbornenes With Vinyl Bromides: Insights From DFT Calculations

**DOI:** 10.3389/fchem.2019.00169

**Published:** 2019-03-27

**Authors:** Fang Ying, Yutong Zhang, Chuyue Xiang, Zhijun Song, Hujun Xie, Weiliang Bao

**Affiliations:** ^1^Department of Applied Chemistry, Zhejiang Gongshang University, Hangzhou, China; ^2^Hangzhou Environmental Monitoring Center Station, Hangzhou, China; ^3^Department of Chemistry, Zhejiang University, Hangzhou, China

**Keywords:** Pd catalysis, DFT calculation, cycloaddition, mechanism, protonation

## Abstract

DFT calculations were performed to elucidate mechanistic details of an unusual palladium-catalyzed methylcyclopropanation from [2 + 1] cycloadditions of (*Z*)-2-bromovinylbenzene and endo-N-(p-tolyl)-norbornenesuccinimide. The reaction proceeds via oxidative addition (OA), intermolecular alkene insertion, deprotonation/protonation, intramolecular alkene insertion, β-H elimination and reductive elimination (RE). Protonation is the rate-limiting step and requires an overall barrier of 28.5 kcal/mol. The sources of two protons for protonation and exchange have also been clarified and the calculations agree with experimental observations.

## Introduction

Cyclopropane skeleton has attracted tremendous attention from organic chemists and can be found in many important biomolecules and pharmaceutical drugs (Hofmann et al., 1954; Crowley et al., 1961; Wiberg, 1996; de Meijere, 2003; Fedorynski, 2003; Lebel et al., 2003; Pietruszka, 2003; Reissig and Zimmer, 2003; Wessjohann et al., 2003; Hata et al., 2011; Chen et al., 2014; Hiratsuka et al., 2014). Many methods have been used to construct the cyclopropane scaffold, including transition metal mediated C–C and C–H bond activations (Satake and Nakata, 1998; Goudreau and Charette, 2010; Oonishi et al., 2012; Masutomi et al., 2014; Du et al., 2015), carbene/carbenoid cycloadditions (Miki et al., 2002; Biswas et al., 2012; Lindsay et al., 2013), Simmons–Smith reactions (Simmons and Smith, 1958; Beaulieu et al., 2013), Michael-initiated ring closure (MIRC) (Xie et al., 2007; Xuan et al., 2009), cycloisomerizations (Bruneau, 2005; Miege et al., 2011), and the coupling of norbornenes with organoboron reagents or alkynes (Bigeault et al., 2005; Miura et al., 2006).

**Scheme 1:**
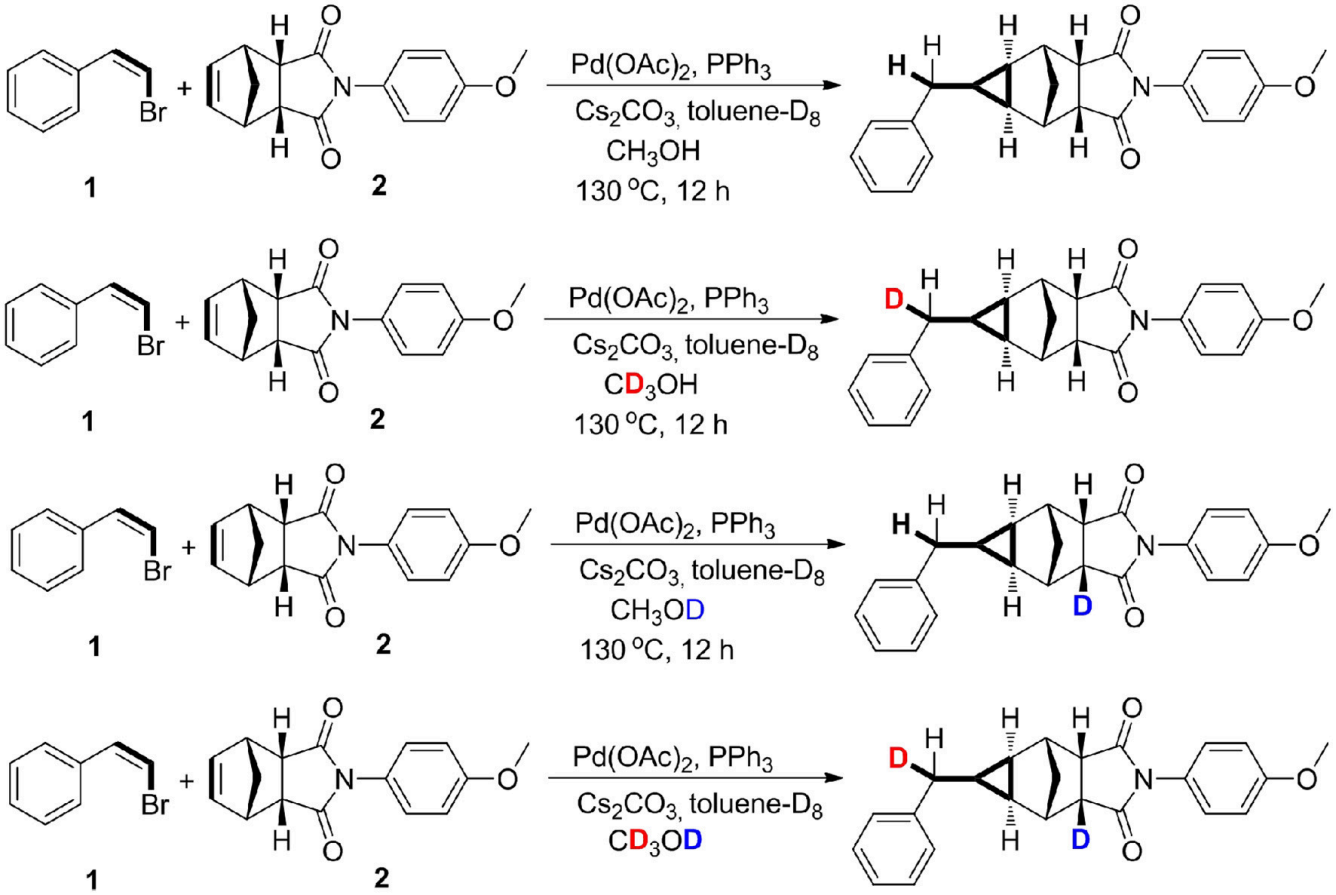
Deuterium-labeling studies.

However, the cyclopropanation of halohydrocarbon with alkenes catalyzed by transition metal catalysts by a non-carbene mechanism is still underdeveloped (Mao and Bao, 2014a; Mao et al., 2014). Recently, we firstly reported the palladium-catalyzed methylcyclopropanation of bromostyrenes with norbornenes via [2 + 1] cycloaddition, and the reactions proceed by a methylene protonation and a H/D exchange with CD_3_OD (Mao et al., 2015). A methylcyclopropane group was constructed through a three-fold domino method including an important protonation process. The experimental results demonstrated that a norbornenylpalladium intermediate could capture one proton from research systems (Palucki et al., 1997; Torraca et al., 2000; Kuwabe et al., 2001; Matsukawa et al., 2005; Tseng et al., 2006; Dash and Janni, 2012; Mao and Bao, 2014b). The mechanistic studies revealed that the methylcyclopropanation step proceeds via a protonation and a H/D exchange with CD_3_OD. As shown in Scheme 1, two different deuterium atoms from CD_3_OD were chemoselectively added into the two positions of methylcyclopropane derivatives. Herein, quantum chemistry (QC) calculations have been used to elucidate the reaction mechanisms, and the protonation step and a H/D exchange process from CD_3_OD have also been explored and discussed.

**Graphical Abstract:**
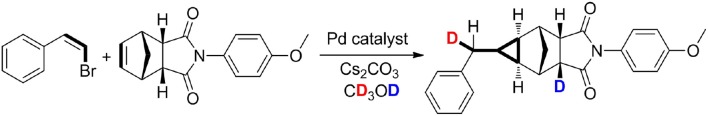
Palladium-Catalyzed Methylcyclopropanation of Norbornenes With Vinyl Bromides.

## Computational Methods

**Figure 1:**
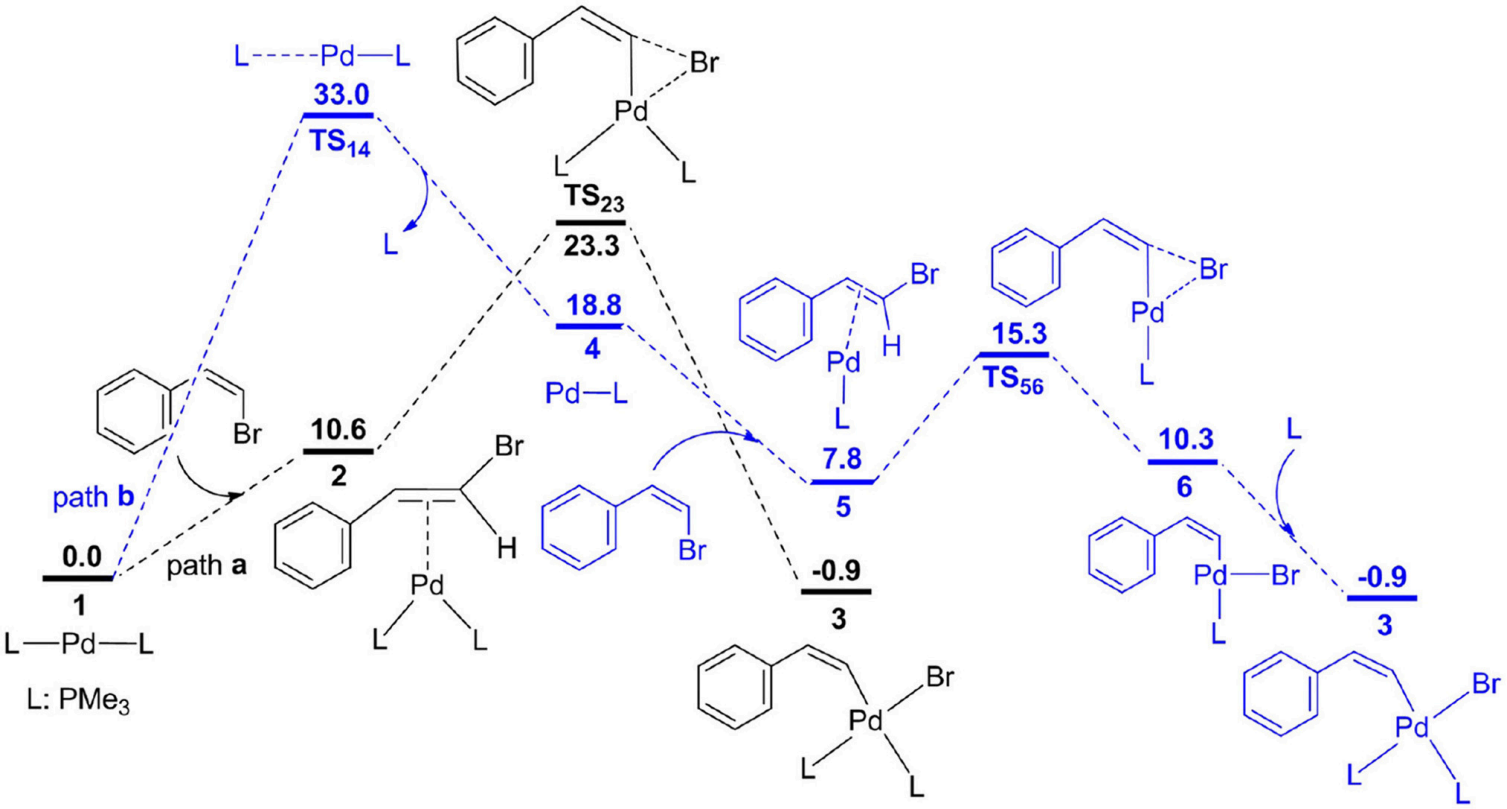
Free energy profiles for two possible oxidative addition pathways.

All of species were optimized through M06 functional (Zhao and Truhlar, 2006a,b, 2008) in combination with 6-31G(d,p) basis set for H, C, O and N atoms. The Pd, P, Br, and Cs atoms were described by LANL2DZ basis set (Ehlers et al., 1993; Check et al., 2001). The polarization functions involving Pd(ζ_f_) = 1.472 (Huzinaga, 1984), Br(ζ_d_) = 0.389, P(ζ_d_) = 0.340, and Cs(ζ_f_) = 0.306 were also added (Amatore et al., 1992). The structural parameters of complex **1** from calculations are consistent with the measured parameters from experiments (Figure 1; Mao et al., 2015) suggesting that the computational method in our calculations is right. Frequency analyses have been used to obtain the zero-point energies (ZPE), and then confirmed the transition states with only one imaginary frequency and the intermediates with zero imaginary frequency. Each transition state was also validated through intrinsic reaction coordinate calculations to connect the reactant and product (Fukui, 1970, 1981). Natural bond orbital (NBO) was carried out to obtain atomic charge distribution (Reed and Weinhold, 1985; Reed et al., 1985, 1988). In order to reduce the costs for computation, the triphenylphosphine (PPh_3_) ligand used in experiments was replaced by trimethylphosphine (PMe_3_), and the reliability of this models has been validated by previous calculations (Xie et al., 2013a,b). All calculations were performed by Gaussian09 software (Frisch et al., 2009).

A continuum medium strategy based on the optimized species in gas-phase was performed to obtain single point energy in solvent. We selected the conductor-like polarizable continuum model (CPCM) involving an UAHF radii method (Barone and Cossi, 1998; Cossi et al., 2003). Toluene was utilized as solvent based on reaction conditions.

The entropy change was taken into consideration in a bimolecular process, and the corrections were added to the free energies based on the free volume theory (Benson, 1982). For 2 to 1 (or 1 to 2) change, a correction of −2.6 (or 2.6) kcal/mol was necessary. The corrections have been validated by previous calculations (Okuno, 1997; Ardura et al., 2005; Liu et al., 2009, 2012; Schoenebeck and Houk, 2010; Wang et al., 2012a,b). The relative Gibbs free energies from solvent were adopted to analyze the reaction mechanisms in this manuscript.

## Results and Discussion

**Figure 2:**
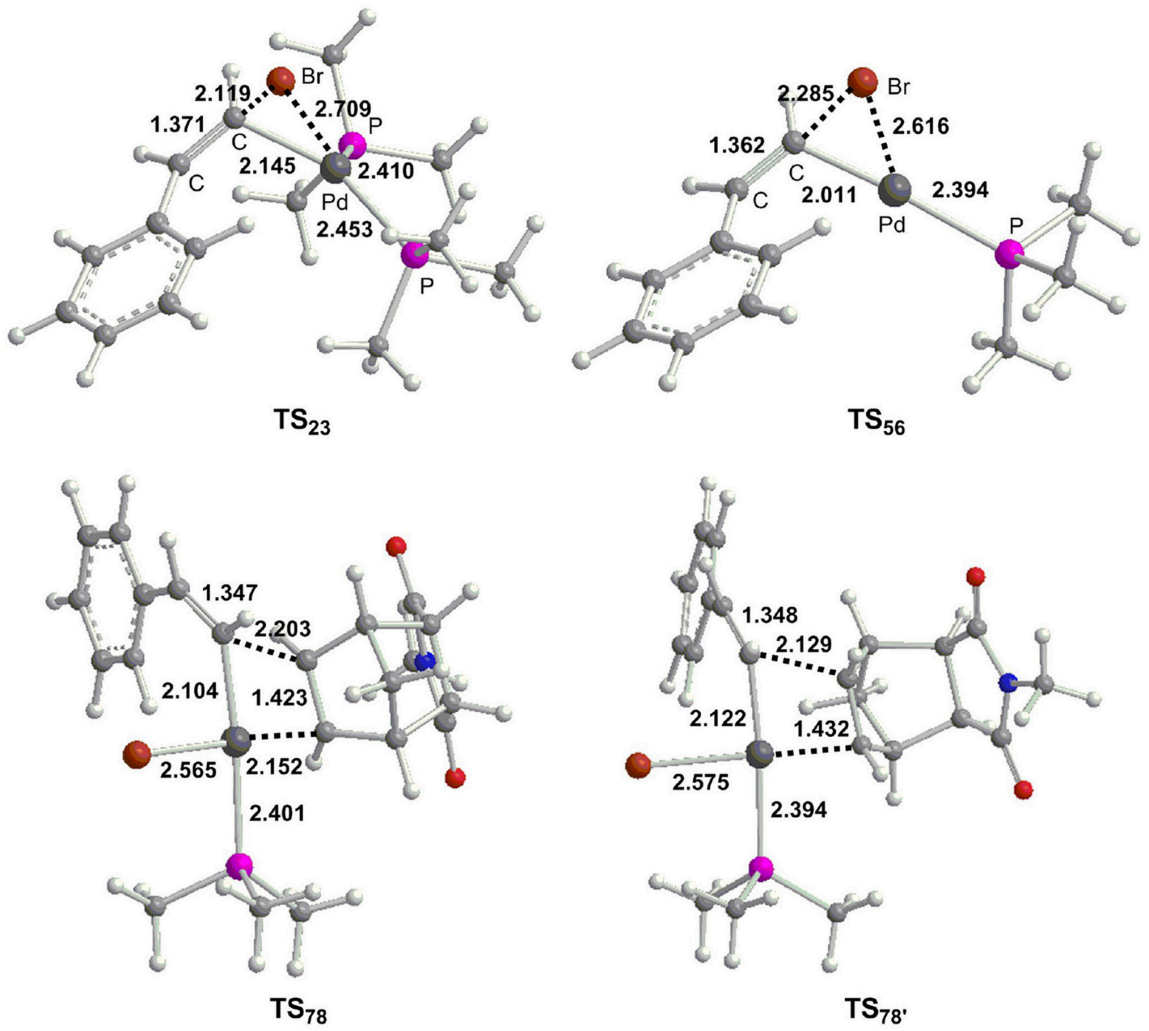
Optimized geometries (Å) for selected transition states.

Oxidative addition is expected to be the initial step for Pd-catalyzed methylcyclopropanation of norbornene with vinyl bromide, and the corresponding free energy profiles are shown in Figure 1, and optimized geometries for different transition states are described in Figure 2. From palladium bisphosphine complex **1**, two possible pathways for the formation of complex **3** are proposed. Path **a** (black) is related to the bisphosphine pathway and path **b** (blue) involves the monodentate phosphine pathway. The calculation results showed that path **a** is preferred. In path **a**, the double bond of substrate (*Z*)-2-bromovinylbenzene is coordinated to the Pd center to produce complex **2**, and the process is endergonic via 10.6 kcal/mol. Subsequently, the three-membered ring oxidative addition transition state has been located with an overall barrier of 23.3 kcal/mol from **1** to **TS**_23_, and generates a square-planar complex **3**. In path **b**, one phosphine ligand of complex **1** is dissociated to give complex **4**, and the barrier is predicted to be 33.0 kcal/mol for dissociation process based on the method proposed by Hall and coworkers (Hartwig et al., 2005). From **4**, the substrate enters into reaction system to yield complex **5**, followed by oxidative addition with a barrier (**TS**_56_) of 7.5 kcal/mol to afford a three-coordinate complex **6**. Finally, complex **3** is produced via the coordination of phosphine ligand.

**Figure 3:**
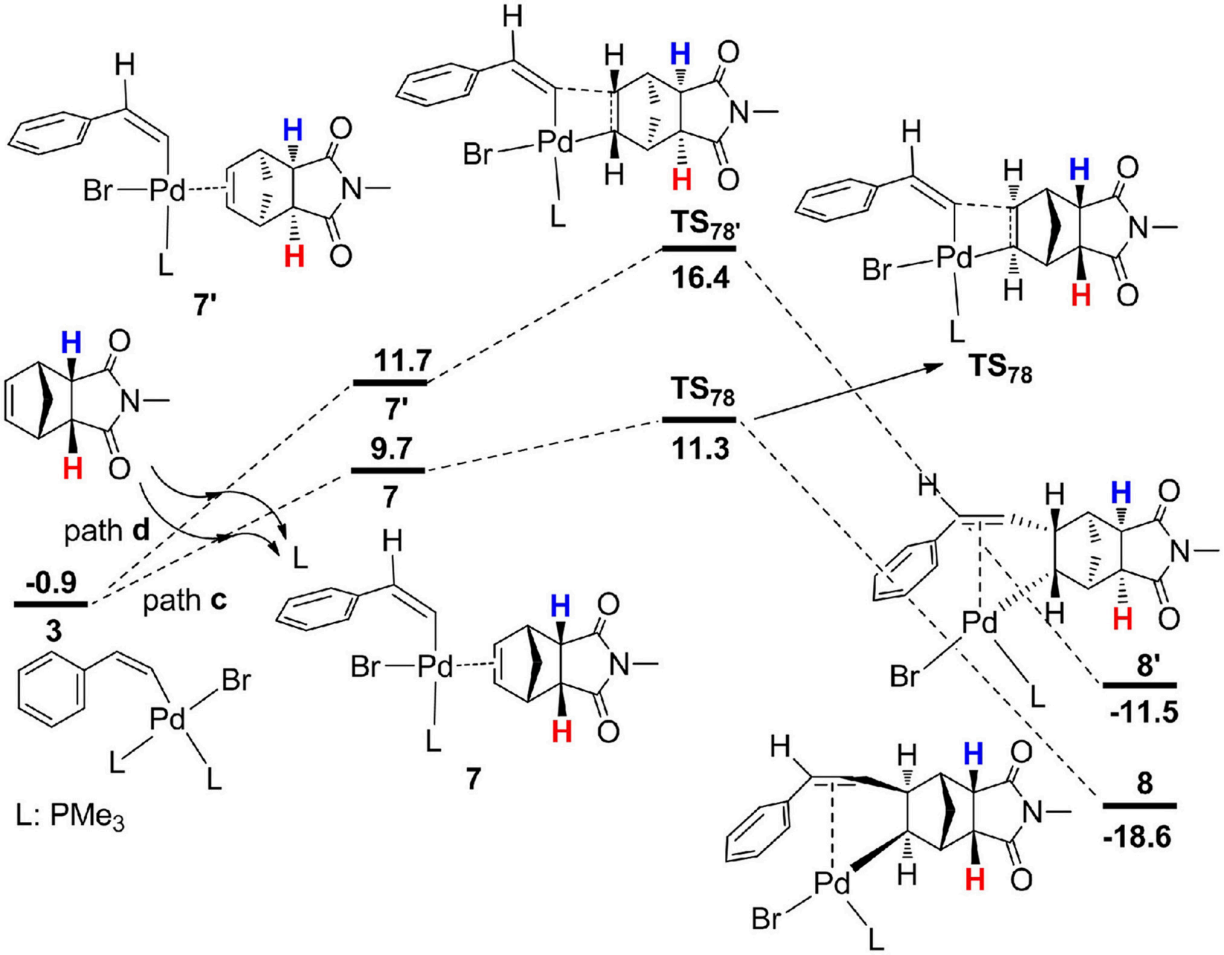
Free energy profiles for two possible intermolecular alkene insertion pathways.

From **3**, the reaction proceeds by intermolecular alkene insertion step, and two possible pathways are presented considering different coordination directions of endo-N-(p-tolyl)-norbornenesuccinimide (Figure 3). In path **c**, two bridge-hydrogen atoms and the bridge-carbon atom of norbornene moieties are outside of the plane. While in path **d**, two bridge-hydrogen atoms and the bridge-carbon atom of norbornene moieties locate inside of the plane. According to the calculations, path **c** (12.2 kcal/mol for **TS**_78_) is more favorable than path **d** (17.3 kcal/mol for **TS**${}_{78^{\text{′}}}$) by 5.1 kcal/mol, then a stable four-coordinate intermediate **8** is formed and this process is obviously exergonic by 17.7 kcal/mol.

**Figure 4:**
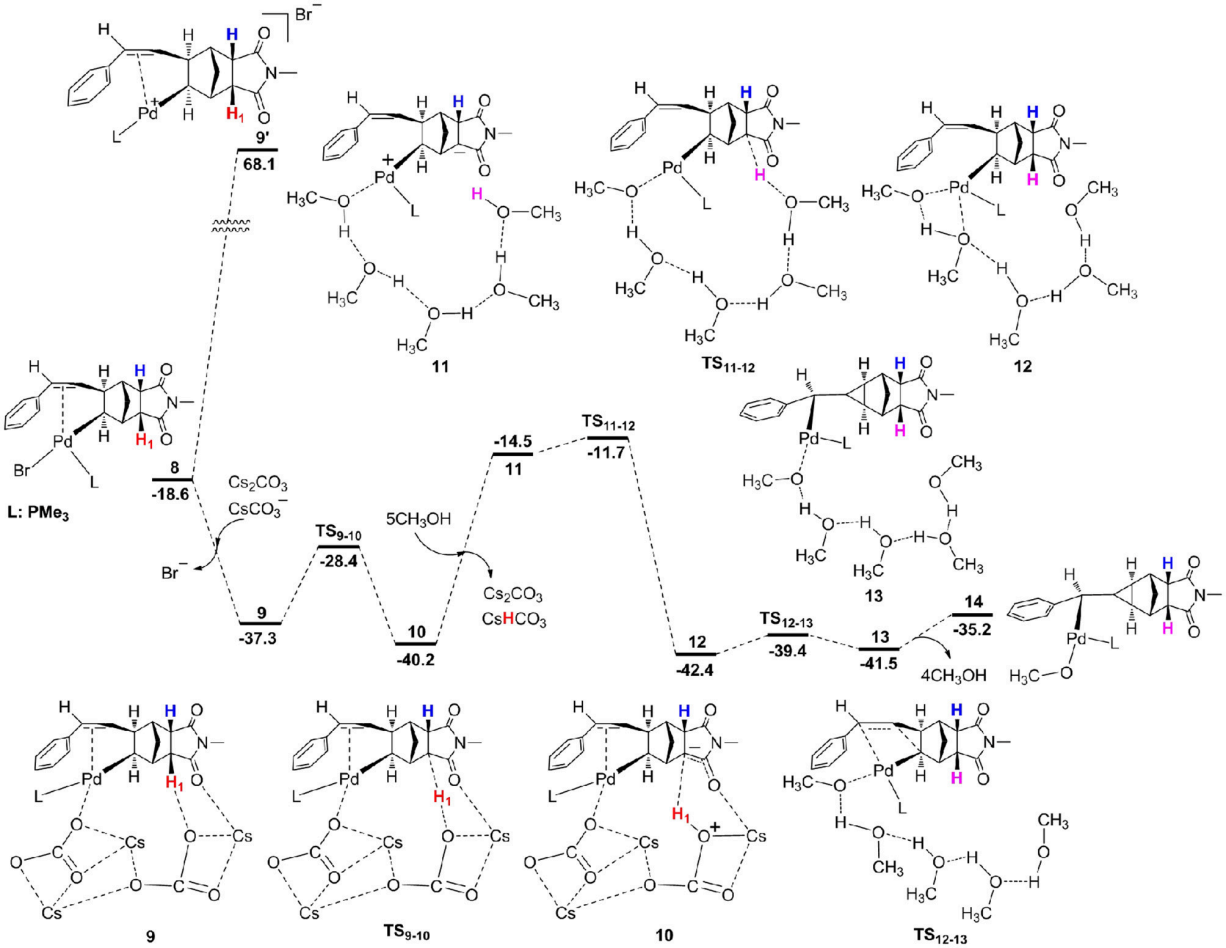
Free energy profiles for proton exchange with CH_3_OH and intramolecular alkene insertion.

**Figure 5:**
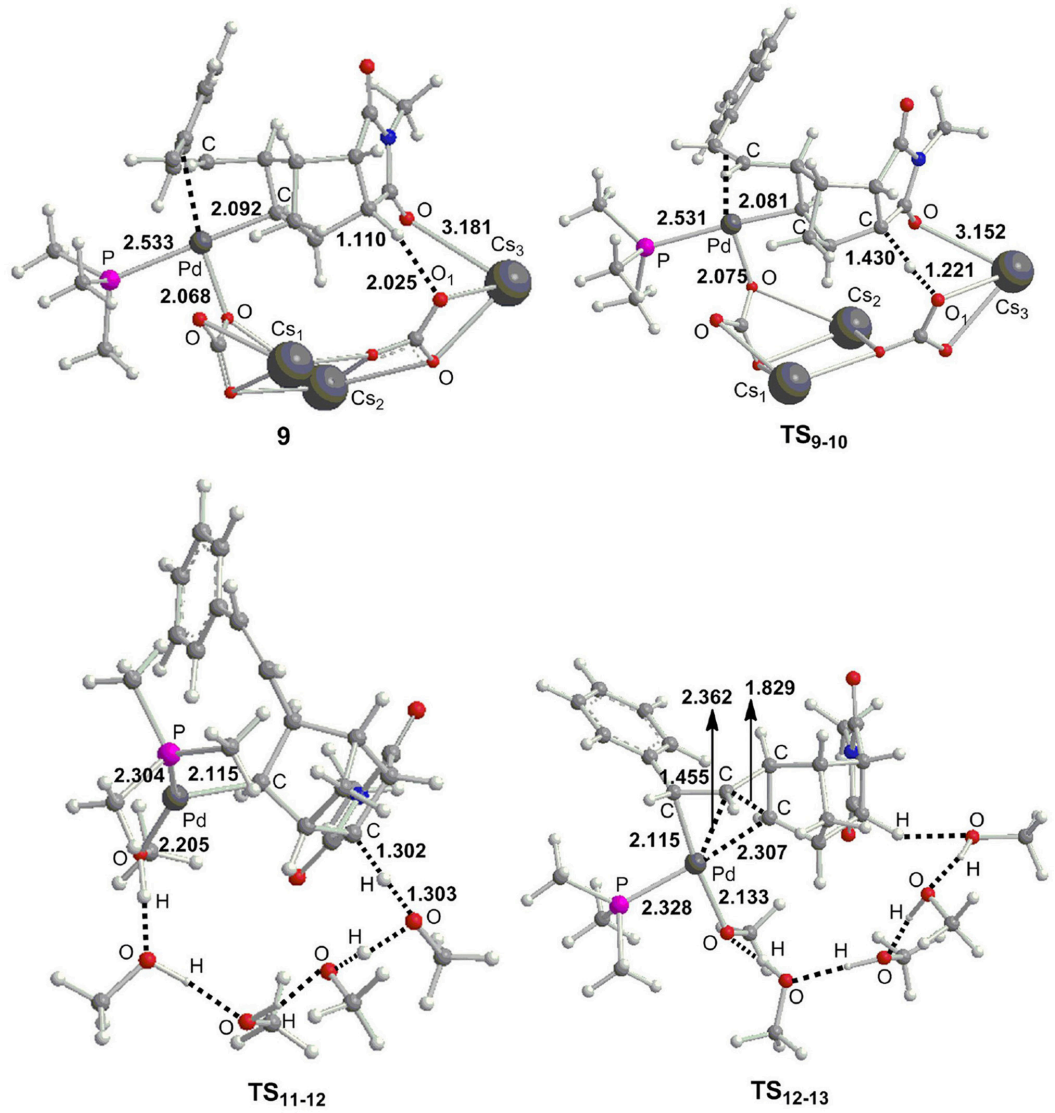
Optimized important geometries (Å) as presented in Figure 4.

From **8**, we consider the possibility for the formation of ion pair complex **9'** as described in previous experiments (Mao et al., 2015). The calculations showed the relative Gibbs free energy of this complex is very high with a value of 68.1 kcal/mol (Figure 4), thus we exclude this possibility. Alternatively, we investigate the key role of base in deprotonation, which has been confirmed in previous experiments (Wasa et al., 2009; Liang et al., 2012) and calculations (Biswas et al., 2000; Davies et al., 2005; Lafrance et al., 2007; Ess et al., 2008; Kefalidis et al., 2010; Figg et al., 2013; Xie et al., 2013c, 2016). However, it is interesting to note that the γ-H_1_ in complex **8** is far away from palladium center with the Pd–H_1_ distance of 5.268 Å (Figure 4), therefore, it is very difficult to activate this C–H_1_ bond. The γ-C–H activation has been previously accomplished by Yu et al. (Li et al., 2014; Jiang et al., 2016; Wu et al., 2016; Shao et al., 2017, 2018; Zhu et al., 2018), and they developed a weakly coordinating directing group to help the C–H bond activation. From **8**, the ligand substitution of Cs_2_CO_3_ and CsCO${}_{3}^{-}$ for Br^−^ occurs to give a stable complex **9**, where the γ-H_1_ generates weak hydrogen bond interaction with the oxygen atom of CsCO${}_{3}^{-}$. The γ-C–H_1_ distance is 1.110 Å in complex **9** (Figure 5), indicating that this bond has been activated. Subsequently, the deprotonation is easy to take place to give complex **10** with a barrier (**TS**_9−10_) of only 8.9 kcal/mol. The C–H_1_ and O–H_1_ bond length in **TS**_9−10_ are 1.430 Å and 1.221 Å, respectively (Figure 5). For comparison, the α-H and β-H on the same side of Pd center can be activated by palladium center, and the barriers for α-H (26.8 kcal/mol) and β-H (14.2 kcal/mol) are much higher than that of γ-H. From **10**, the ligand substitution of five CH_3_OH molecules for Cs_2_CO_3_ and CsHCO_3_ takes place to generate an unstable complex **10**, and this process is significantly endergonic by 25.7 kcal/mol, accompanied by protonation via **TS**_11−12_ to yield complex **12**. It is worth noting that the proton comes from hydroxyl of methanol. An overall barrier of protonation step is 28.5 kcal/mol from **10** to **TS**_11−12_, which is the rate-limiting step of catalytic cycle. We have used several density functionals including B3LYP-D3 (Becke, 1993; Stephens et al., 1994), TPSS (Tao et al., 2003), M06-2X (Zhao and Truhlar, 2008), WB97X-D (Chai and Head-Gordon, 2008) to evaluate the functional dependency of this transition metal system. The calculations demonstrated that different functionals have slight effect on the rate-determining state. The barriers (**TS**_11−12_) for B3LYP-D3, TPSS, M06-2X, and WB97X-D are 26.9, 31.2, 29.3, and 27.8 kcal/mol, respectively. From **12**, intramolecular alkene insertion occurs to give a cyclopropanepalladium complex **13** and it requires a barrier (**TS**_12−13_) of only 3.0 kcal/mol. Then complex **14** is generated via the release of four methanol molecules. We know that the γ-H_1_ in complex **8** is far away from palladium center, thus five CH_3_OH molecules are necessary to form the hydrogen bonding network between γ-H_1_ and Pd center for proton transfer in **TS**_11−12_. In addition, we also considered the influence of methanol number on the barriers for proton transfer, and the calculations showed that it has only slight effect. The barriers are 28.5 kcal/mol (**TS**_11−12_) for five methanol molecules, 31.4 kcal/mol (**TS**_11−12__A) for six methanol molecules, 30.9 kcal/mol (**TS**_11−12__B) for seven methanol molecules, and 30.3 kcal/mol (**TS**_11−12__C) for eight methanol molecules, respectively (see Supporting Information). We also consider the other possible pathway for proton exchange with CH_3_OH and intramolecular alkene insertion, where the intramolecular alkene insertion occurs first (see Figure S1). The calculations illustrated that the protonation by methanol molecule is the rate-determining step for catalytic cycle, and needs much higher overall barrier (35.8 kcal/mol from **11′** to **TS**${}_{12-13^{\prime }}$) than the barrier mentioned above (28.5 kcal/mol from **10** to **TS**_11−12_).

**Figure 6:**
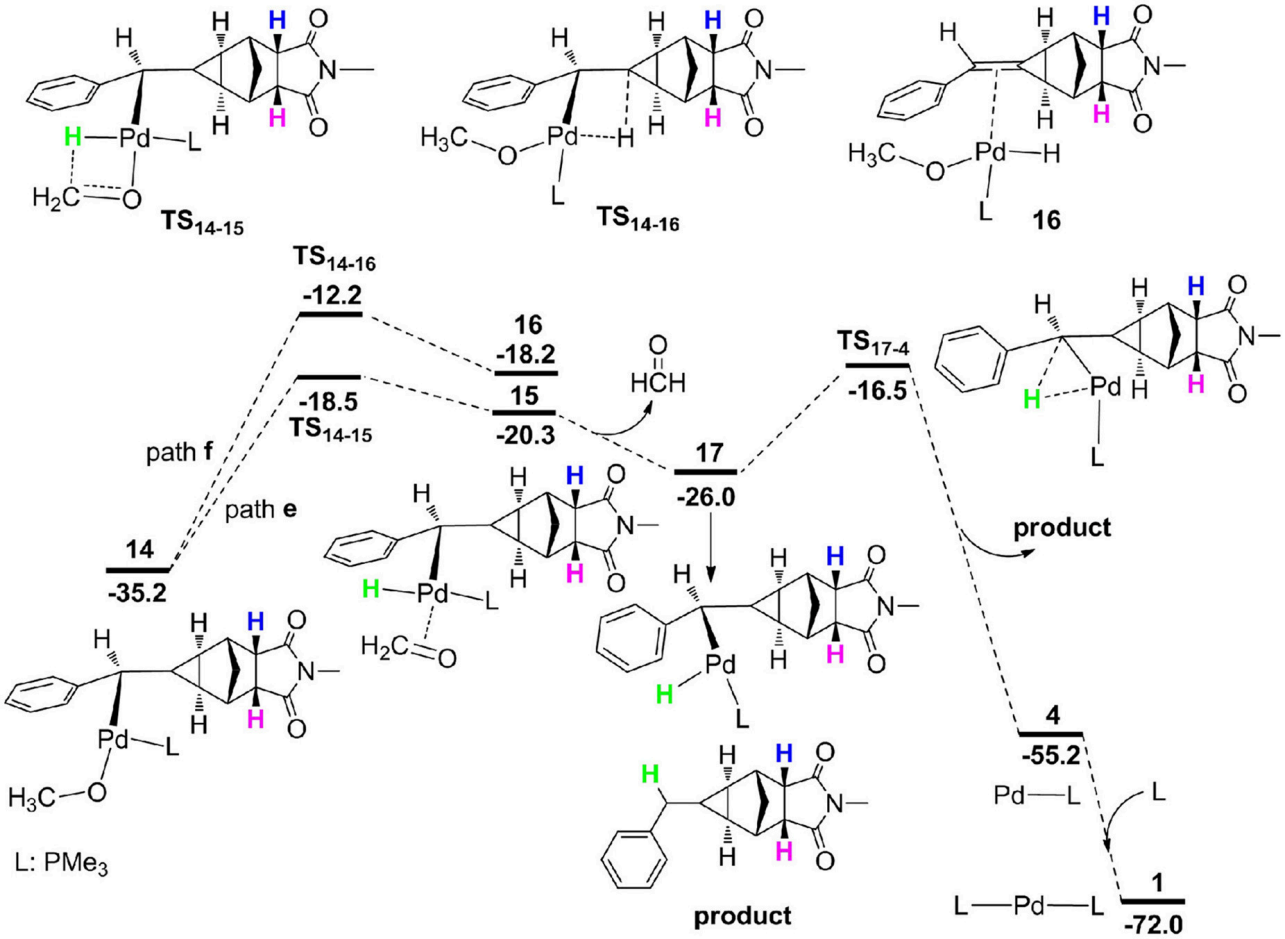
Free energy profiles for β-H elimination and C–H bond reductive elimination.

**Figure 7:**
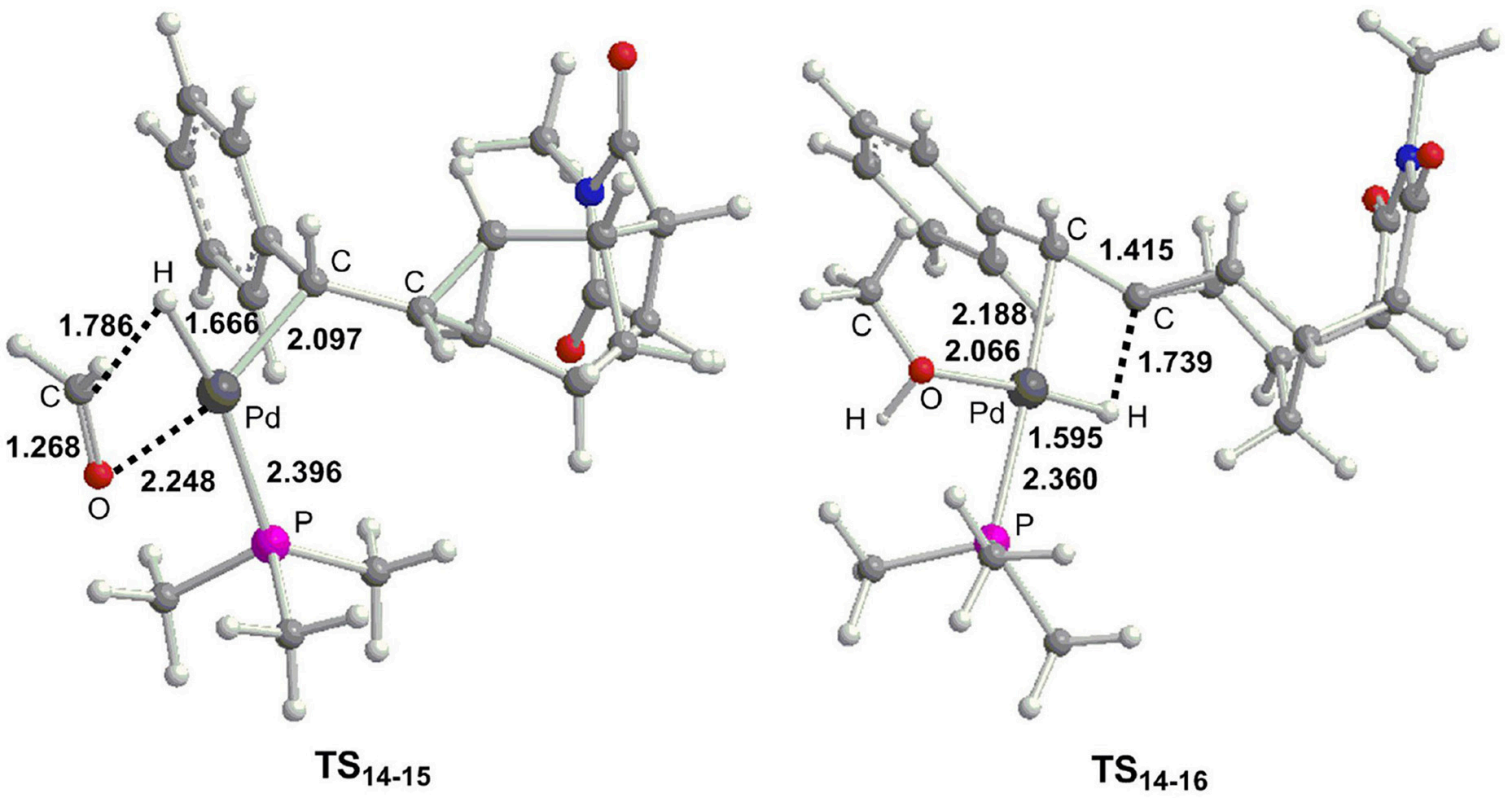
Optimized geometries (Å) for selected transition states as presented in Figure 6.

From **14**, the reaction can proceed via β-H elimination and two possible pathways are proposed due to the existence of two β-H atom for Pd center (Figure 6). One is from methoxyl group (path **e**) and the other is from the cyclopropane carbon-bonded hydrogen atom (path **f**). The calculations demonstrated that path **e** (16.7 kcal/mol for **TS**_14−15_) is more favorable than path **d** (23.0 kcal/mol for **TS**_14−16_), and optimized geometries of two transition states are described in Figure 7. Subsequently, a square-planar complex **15** is generated, followed by the release of methanal to produce complex **17**. A methylcyclopropane product is then formed via the C–H bond reductive elimination, and it needs a barrier (**TS**_17−4_) of 9.5 kcal/mol. Finally, one phosphine ligand is coordinated to the Pd center to regenerate the catalyst. It is clearly to see that the proton for the protonation of a methylcyclopropane subunit comes from the methyl of CH_3_OH, which is consistent with the deuterium-labeling experiments (Fedorynski, 2003).

**Figure 8:**
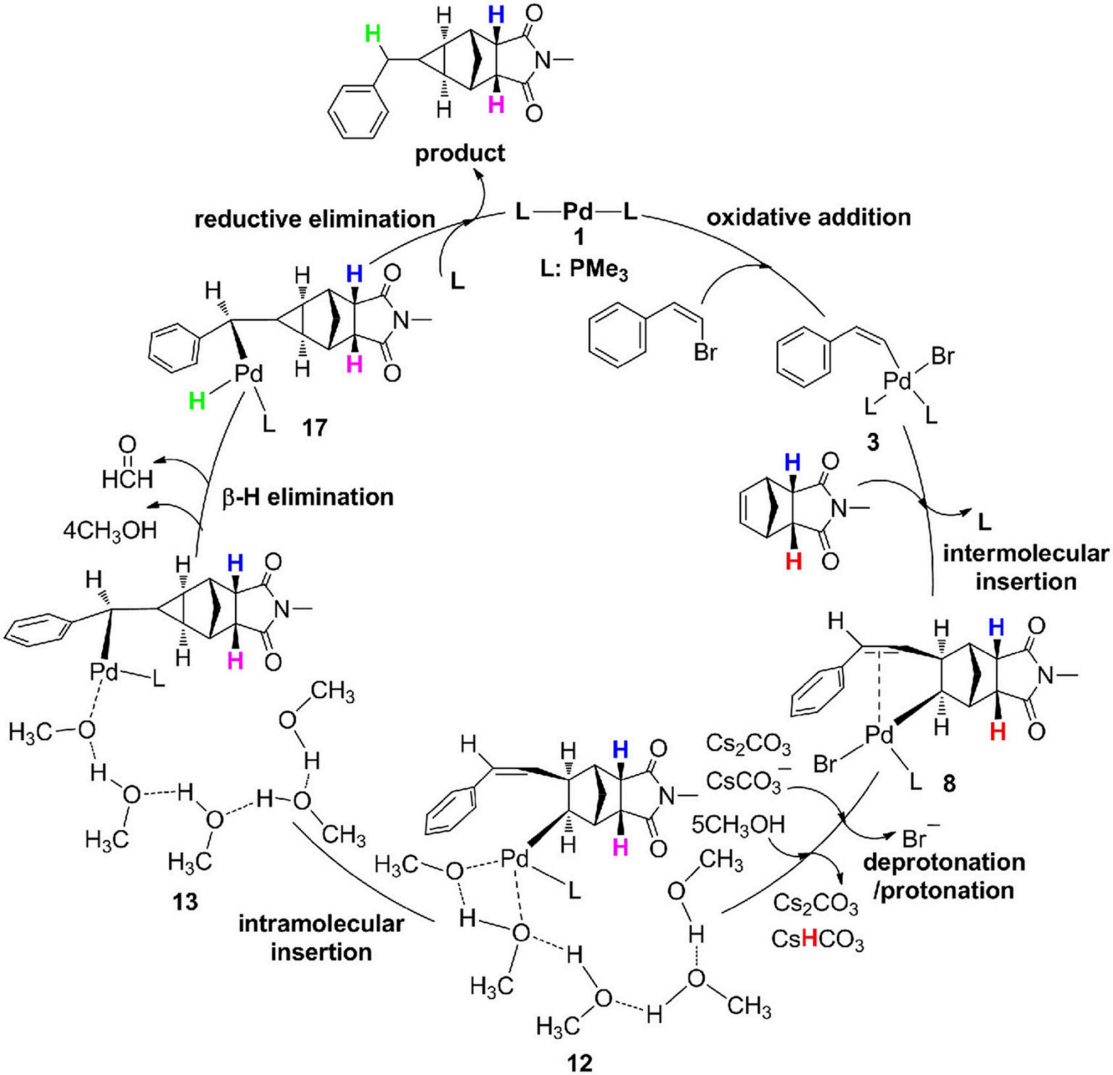
Catalytic cycle for palladium-catalyzed methylcyclopropanation between (*Z)*-2-bromovinylbenzene and endo-N-(p-tolyl)-norbornenesuccinimide.

As described in Figure 8, the catalytic cycle for the reaction of (*Z*)-2-bromovinylbenzene with endo-N-(p-tolyl)-norbornenesuccinimide undergoes six steps, consist of oxidative addition (OA), intermolecular olefin insertion, deprotonation/protonation, intramolecular olefin insertion, β-H elimination and reductive elimination (RE), and protonation is the rate-determining step and requires an overall barrier of 28.5 kcal/mol from **10** to **TS**_11−12_.

## Conclusions

In conclusion, Pd-catalyzed [2 + 1] cycloaddition domino reaction mechanisms of (*Z)*-2-bromovinylbenzene and endo-N-(p-tolyl)-norbornenesuccinimide have been studied by DFT calculations. The results revealed that the methylcyclopropanation process underwent six steps, including oxidative addition, intermolecular alkene insertion, deprotonation/protonation, intramolecular alkene insertion, β-H elimination and reductive elimination, and protonation by methanol is the rate-limiting step with an overall barrier of 28.5 kcal/mol. In addition, the hydrogen atoms for protonation and exchange are both from the methanol, and the former comes from the methyl of methanol, and the latter comes from the hydroxyl of methanol. These calculation results are consistent with the deuterium-labeling experiments.

## Author Contributions

The work was completed by cooperation of all authors. HX and WB were responsible for the study of concept and design of the project. FY, YZ, CX, and ZS searched the intermediates and transition states and analyzed the data and drew energy profiles. FY, YZ, HX, and WB drafted and revised the manuscript.

### Conflict of Interest Statement

The authors declare that the research was conducted in the absence of any commercial or financial relationships that could be construed as a potential conflict of interest.
