# Supplementary material for: Key Mechanistic Features in Palladium-Catalyzed Methylcyclopropanation of Norbornenes With Vinyl Bromides: Insights From DFT Calculations
Source: Front Chem. 2019 Mar 27;7:169. doi: 10.3389/fchem.2019.00169 (PMC6445852; doi:10.3389/fchem.2019.00169)
Supplement: Supplementary file 1 [file Table_1.DOC]

**Supporting Information**

**Key Mechanistic Features in Palladium-Catalyzed Methylcyclopropanation of Norbornenes with Vinyl Bromides: Insights from DFT Calculations**

Fang Ying^1,2^ Yutong Zhang^1^, Chuyue Xiang^1^, Zhijun Song^1^, Hujun Xie^1,*^ Weiliang Bao^3^

^1^Department of Applied Chemistry, Zhejiang Gongshang University, Hangzhou 310018, People’s Republic of China.

*^2^Hangzhou Environmental Monitoring Center Station, Hangzhou 310007, China.*

^3^Department of Chemistry, Zhejiang University, Hangzhou 310027, People’s Republic of China.

***TABLE OF CONTENT***

I.

optimized Structures of TS11-12_A, TS11-12_B and TS11-12_C S2-S4

II.

Free energy profiles for intramolecular alkene insertion and proton exchange with CH3OH S5

III.

Cartesian coordinates, absolute electronic energies and free energies from gas phase for all structures S6-S26


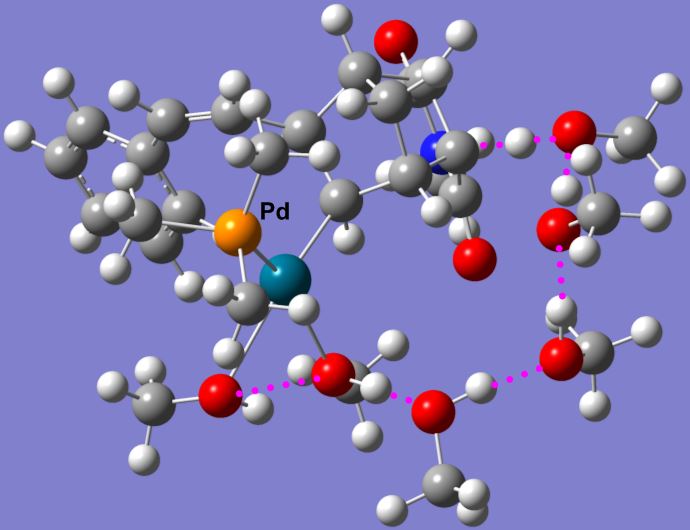


**TS11-12_A**


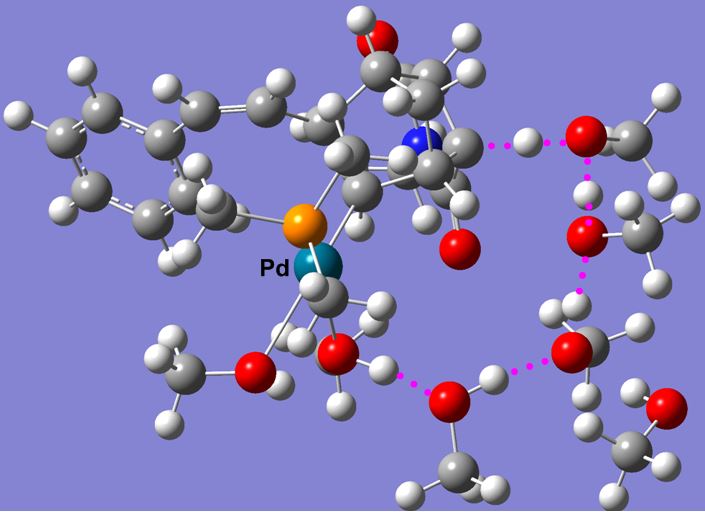


**TS11-12_B**


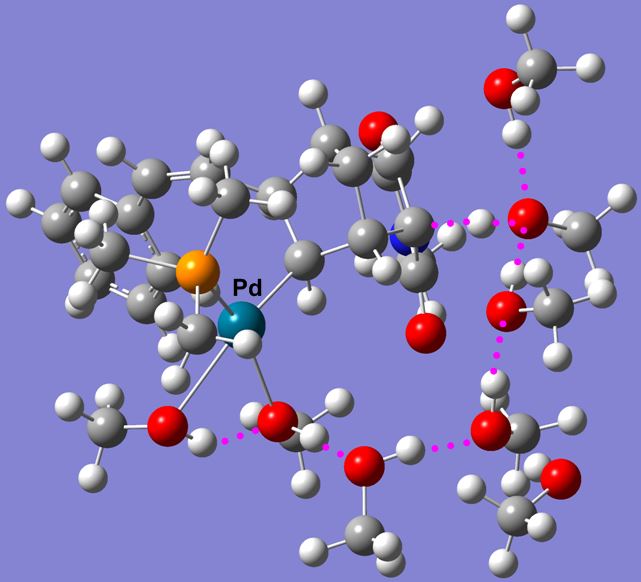


**TS11-12_C**

**Figure S1.** Free energy profiles for intramolecular alkene insertion and proton exchange with CH_3_OH.

# Cartesian coordinates and electronic energies for all of the calculated structures

**1**

SCF done: -379.329547

thermal Free Energies -379.144907

Pd 0.000009 -0.000128 0.000211

P -2.324704 -0.000118 -0.000472

P 2.324748 0.000194 0.000421

C 3.184433 -1.058642 1.278628

H 2.873858 -2.100049 1.151399

H 4.276184 -0.994376 1.194464

H 2.880324 -0.734986 2.278618

C 3.181718 -0.578742 -1.556782

H 2.873883 0.054442 -2.394342

H 4.273698 -0.543276 -1.459305

H 2.872887 -1.604934 -1.777623

C 3.182591 1.637878 0.276295

H 2.874625 2.047851 1.242874

H 4.274470 1.535194 0.258769

H 2.874245 2.341447 -0.502882

C -3.182805 -1.636073 -0.285755

H -2.874837 -2.344110 0.489514

H -4.274660 -1.533186 -0.267918

H -2.874731 -2.040680 -1.254559

C -3.182072 0.570232 1.559690

H -2.872828 1.594964 1.786639

H -4.274027 0.535870 1.461537

H -2.874916 -0.067977 2.393678

C -3.183957 1.065869 -1.273020

H -2.879259 0.748010 -2.274686

H -4.275731 1.000822 -1.189754

H -2.873728 2.106615 -1.139678

**2**

SCF done: -701.553654

thermal Free Energies -701.258560

Pd -0.611032 0.318507 -0.449273

P -2.850878 -0.189246 0.267938

P -0.052907 2.628343 -0.014135

C 1.263173 3.357239 -1.119351

H 0.894717 3.388052 -2.149709

H 1.546829 4.369905 -0.809222

H 2.143610 2.708277 -1.088568

C -1.326862 3.996973 -0.046173

H -2.114348 3.777669 0.681390

H -0.884800 4.972344 0.188553

H -1.785962 4.043798 -1.038805

C 0.714168 2.895066 1.667355

H 1.566303 2.217984 1.780034

H 1.051679 3.929472 1.801777

H -0.018168 2.653742 2.444251

C -3.744975 -1.461911 -0.765106

H -3.131656 -2.366396 -0.815840

H -4.725797 -1.712442 -0.343996

H -3.878400 -1.079467 -1.781974

C -2.931156 -0.989748 1.951790

H -2.586331 -0.281672 2.711872

H -3.948437 -1.315102 2.199298

H -2.260060 -1.853675 1.958062

C -4.183169 1.114779 0.429078

H -4.327204 1.609564 -0.536631

H -5.137476 0.684466 0.755551

H -3.869171 1.871073 1.155323

C 4.767849 0.249958 -0.288055

C 3.597440 0.053358 -1.021164

C 2.421925 -0.436363 -0.417638

C 2.470451 -0.718485 0.963148

C 3.642043 -0.527050 1.693649

C 4.798752 -0.041172 1.076438

H 5.657388 0.626759 -0.787084

H 3.590449 0.273037 -2.086861

H 1.580394 -1.081894 1.464160

H 3.649844 -0.757046 2.756445

H 5.708555 0.107896 1.651709

C 1.216099 -0.593877 -1.258981

H 1.351521 -0.205972 -2.270159

C 0.144023 -1.520885 -1.167142

H -0.348032 -1.823866 -2.085762

Br 0.138292 -3.093188 0.026246

**TS23**

SCF done: -701.521897

thermal Free Energies -701.521897

Pd -0.781348 0.022524 0.220926

P -2.528695 -1.309302 -0.770752

P -0.812326 2.419070 -0.299963

C 0.269905 2.886684 -1.749248

H -0.121065 2.414762 -2.656035

H 0.311025 3.972194 -1.898031

H 1.280917 2.502848 -1.582354

C -2.402264 3.292344 -0.757553

H -3.113556 3.209168 0.070196

H -2.234662 4.352795 -0.979783

H -2.844031 2.810433 -1.635454

C -0.167775 3.575762 1.019068

H 0.838487 3.262992 1.313236

H -0.136786 4.615815 0.673368

H -0.810502 3.509165 1.902406

C -2.063846 -2.079775 -2.407836

H -1.152838 -2.671864 -2.277064

H -2.860440 -2.721511 -2.802416

H -1.847407 -1.287876 -3.131414

C -3.149442 -2.814505 0.151253

H -3.595804 -2.500314 1.099781

H -3.893212 -3.372271 -0.429835

H -2.307994 -3.477713 0.375832

C -4.152539 -0.494067 -1.212441

H -3.961014 0.349323 -1.882926

H -4.842993 -1.191319 -1.701723

H -4.621778 -0.105684 -0.303165

C 4.711664 -1.063052 -1.806848

C 3.571723 -1.724473 -1.356148

C 2.626036 -1.092589 -0.518005

C 2.887305 0.248274 -0.154652

C 4.033115 0.903453 -0.596374

C 4.954500 0.258402 -1.428542

H 5.413933 -1.584379 -2.452856

H 3.402979 -2.757932 -1.651668

H 2.182139 0.774071 0.478070

H 4.208218 1.932486 -0.290358

H 5.842585 0.778941 -1.776357

C 1.483782 -1.882920 -0.071596

H 1.474578 -2.895763 -0.482680

C 0.431094 -1.656815 0.777164

H -0.158075 -2.506995 1.105037

Br 0.611574 -0.435267 2.499567

**3**

SCF done: -701.574289

thermal Free Energies -701.276754

Pd -0.563994 0.084641 -0.461261

P 0.168993 2.297457 -0.359578

P -2.557789 0.019529 1.025289

C -3.159231 1.423136 2.107083

H -3.443572 2.282448 1.493659

H -4.029272 1.108876 2.694189

H -2.366644 1.732603 2.795810

C -4.113115 -0.411535 0.102340

H -3.911695 -1.297491 -0.504635

H -4.942543 -0.607000 0.791071

H -4.383529 0.414570 -0.563044

C -2.424658 -1.344208 2.282513

H -1.625508 -1.107400 2.992262

H -3.365850 -1.470995 2.829023

H -2.166110 -2.267280 1.758979

C 1.730043 2.604238 0.595394

H 2.536231 1.996733 0.179769

H 2.002555 3.664732 0.552622

H 1.583382 2.307425 1.637574

C 0.533480 3.059156 -2.014610

H -0.370019 3.041797 -2.631463

H 0.872513 4.094044 -1.897160

H 1.306662 2.470258 -2.511830

C -0.978473 3.571855 0.367926

H -1.161402 3.351535 1.421982

H -0.541243 4.572871 0.283354

H -1.933780 3.553541 -0.164520

C 5.119039 -0.555332 0.934856

C 4.394126 -0.394684 -0.245565

C 3.005978 -0.627634 -0.289790

C 2.375846 -1.067004 0.890426

C 3.100976 -1.229950 2.070124

C 4.473822 -0.968904 2.102562

H 6.189211 -0.363275 0.941353

H 4.905405 -0.077831 -1.152549

H 1.319339 -1.316140 0.860176

H 2.594683 -1.582292 2.965865

H 5.036880 -1.102682 3.022585

C 2.281124 -0.423298 -1.557394

H 2.914478 -0.513218 -2.445818

C 0.981076 -0.149350 -1.758522

H 0.632165 -0.071713 -2.789843

Br -1.137053 -2.353752 -0.995747

**4**

SCF done: -253.025026

thermal Free Energies -252.944054

Pd 1.341110 -0.000110 0.000076

P -0.885797 0.000123 -0.000217

C -1.739829 -1.074621 -1.267773

H -1.430770 -2.114230 -1.126016

H -2.831753 -1.006506 -1.183852

H -1.434346 -0.763005 -2.270757

C -1.740169 -0.560996 1.564106

H -1.436983 0.085047 2.393044

H -2.832062 -0.528799 1.460314

H -1.429625 -1.583213 1.798677

C -1.739125 1.635943 -0.296389

H -1.432122 2.032301 -1.268530

H -2.831129 1.530794 -0.276398

H -1.430561 2.348877 0.473610

**5**

SCF done: -575.271346

thermal Free Energies -575.081873

Pd -0.901444 -0.390372 -0.413796

P -2.934543 0.612351 0.229682

C -4.416678 -0.519220 0.329074

H -4.220803 -1.310770 1.058704

H -5.320671 0.026105 0.624411

H -4.581605 -0.988356 -0.645455

C -3.003248 1.444589 1.898259

H -2.247437 2.234498 1.938076

H -3.991031 1.878312 2.094091

H -2.769999 0.712453 2.677150

C -3.582650 1.965404 -0.879083

H -3.713354 1.571006 -1.891014

H -4.539441 2.360480 -0.517500

H -2.850107 2.776747 -0.922068

C 3.324755 2.951885 -0.615984

C 2.531448 1.987529 -1.234450

C 1.961787 0.928422 -0.501850

C 2.200525 0.882455 0.885568

C 2.993619 1.847388 1.502224

C 3.562720 2.884492 0.757724

H 3.755587 3.755499 -1.207534

H 2.352404 2.046463 -2.305695

H 1.748677 0.097968 1.480441

H 3.163363 1.791892 2.574411

H 4.179398 3.634807 1.245232

C 1.154000 -0.056717 -1.246199

H 0.950269 0.244283 -2.274741

C 0.878962 -1.403026 -1.010887

H 0.595108 -2.043856 -1.839843

Br 1.638365 -2.499855 0.405553

**TS56**

SCF done: -575.254067

thermal Free Energies -575.065754

Pd 0.831429 0.254749 -0.515872

P 2.720319 -1.087372 0.086321

C 2.856261 -2.789755 -0.667343

H 2.882007 -2.703197 -1.757863

H 3.757264 -3.312629 -0.326282

H 1.974650 -3.377557 -0.394206

C 4.395426 -0.372018 -0.314217

H 4.500209 0.596899 0.182949

H 5.205615 -1.034902 0.010596

H 4.473578 -0.210879 -1.393647

C 2.907587 -1.475123 1.900722

H 2.024478 -2.021509 2.245391

H 3.803398 -2.075620 2.096809

H 2.968384 -0.538567 2.462901

C -4.668809 -1.715682 -0.345226

C -3.830971 -0.912226 -1.114986

C -2.674394 -0.320066 -0.567473

C -2.391068 -0.563912 0.792858

C -3.232946 -1.362843 1.560964

C -4.374364 -1.944945 0.999846

H -5.552297 -2.160372 -0.795758

H -4.070466 -0.734599 -2.161090

H -1.509242 -0.123367 1.243227

H -2.995717 -1.534105 2.607881

H -5.025191 -2.569949 1.605340

C -1.868973 0.529817 -1.445178

H -2.277291 0.621222 -2.455611

C -0.738918 1.266399 -1.260363

H -0.427909 2.001866 -1.998484

Br -0.201570 2.335186 0.686444

**6**

SCF done: -575.265594

thermal Free Energies -575.077963

Pd -0.495783 -0.211688 -0.718691

P -2.316928 1.345440 -0.085437

C -2.550176 2.982644 -0.947894

H -2.707139 2.813012 -2.017673

H -3.408975 3.530255 -0.544143

H -1.649543 3.592686 -0.826816

C -3.998178 0.566746 -0.239127

H -3.988562 -0.383121 0.302755

H -4.781611 1.216995 0.165493

H -4.209071 0.358736 -1.292538

C -2.269134 1.838878 1.707011

H -1.374675 2.440111 1.896746

H -3.157864 2.413005 1.991281

H -2.208027 0.930275 2.312562

C 4.958275 1.132249 -0.046495

C 4.221729 0.256262 -0.843388

C 2.960757 -0.212127 -0.434094

C 2.473171 0.198308 0.821600

C 3.211627 1.070434 1.618398

C 4.454040 1.546866 1.187303

H 5.927964 1.485832 -0.387188

H 4.621698 -0.066281 -1.802176

H 1.526305 -0.196740 1.178593

H 2.822520 1.368682 2.588682

H 5.027978 2.224336 1.813881

C 2.225264 -1.138131 -1.313291

H 2.858407 -1.656036 -2.042064

C 0.909805 -1.414245 -1.375926

H 0.532216 -2.244059 -1.974143

Br -1.081565 -1.978255 0.919243

**PMe3**

SCF done: -126.254790

thermal Free Energies -126.170964

C -2.662736 1.676712 0.712584

P 0.000172 -0.000318 -0.617206

C 1.533341 -0.607403 0.286492

H 2.395119 0.002722 -0.004405

H 1.420100 -0.561926 1.376492

H 1.743845 -1.642324 -0.003623

C -0.240433 1.631371 0.286086

H -1.200721 2.071842 -0.002660

H -0.220717 1.510654 1.376073

H 0.549427 2.331763 -0.005617

C -1.293118 -1.023569 0.286366

H -1.195363 -2.075509 -0.002425

H -1.198817 -0.946013 1.376363

H -2.294196 -0.688834 -0.005763

**7**

SCF done: -1168.161554

thermal Free Energies -1167.786784

Pd 1.125736 -0.258178 -0.362661

P 1.971939 -2.035835 1.145090

C 0.848435 -3.115876 2.173762

H 0.167980 -3.683655 1.532986

H 1.437029 -3.819087 2.772686

H 0.248493 -2.499569 2.850594

C 3.016769 -3.296785 0.269150

H 3.790533 -2.769569 -0.293708

H 3.476203 -3.989758 0.982213

H 2.394928 -3.860812 -0.432895

C 3.112743 -1.359007 2.446043

H 2.545095 -0.720368 3.130022

H 3.581454 -2.169314 3.015111

H 3.876498 -0.753326 1.953438

C -0.742848 5.215139 0.883675

C -0.416926 4.517560 -0.277778

C 0.287383 3.299151 -0.226872

C 0.679424 2.821549 1.038742

C 0.353533 3.518916 2.201440

C -0.362898 4.716532 2.132119

H -1.292910 6.150176 0.814231

H -0.718072 4.912530 -1.245589

H 1.277189 1.915881 1.097300

H 0.676981 3.134901 3.166168

H -0.610997 5.261168 3.039282

C 0.585400 2.591510 -1.483357

H 0.551082 3.229942 -2.371201

C 0.860549 1.295162 -1.686340

H 1.058750 0.948492 -2.700287

Br 3.572492 0.208472 -0.822711

C -3.249980 -0.799120 -1.590039

C -1.793571 -0.284660 -1.910535

C -1.427310 -2.222344 -0.783440

C -2.998034 -2.117047 -0.810225

H -3.798983 -0.938502 -2.526194

H -3.423632 -3.002533 -1.292674

C -1.116571 -1.648549 -2.186449

H -0.045010 -1.561243 -2.388573

H -1.598783 -2.210877 -2.993438

C -1.180520 0.094793 -0.570273

H -1.318095 1.066422 -0.112254

C -0.955289 -1.086126 0.120794

H -0.955139 -1.166604 1.201483

H -1.750453 0.477887 -2.686424

H -1.056838 -3.221380 -0.551232

C -4.086554 0.064088 -0.655046

C -3.672892 -1.912123 0.539956

O -4.521312 1.177715 -0.861840

O -3.689875 -2.672910 1.487844

N -4.283899 -0.659116 0.528582

C -5.015221 -0.132689 1.670663

H -5.785332 -0.844964 1.977428

H -4.337692 0.034801 2.513495

H -5.467428 0.811499 1.364949

**TS78**

SCF done: -1168.158316

thermal Free Energies -1167.783035

Pd 1.147869 -0.274291 -0.301420

P 2.163192 -1.892951 1.153161

C 1.096867 -2.997550 2.209635

H 0.435626 -3.602024 1.582610

H 1.716090 -3.664469 2.818909

H 0.475495 -2.389267 2.873967

C 3.253983 -3.107429 0.271261

H 3.986397 -2.544924 -0.312857

H 3.764235 -3.767006 0.981656

H 2.648742 -3.711112 -0.412001

C 3.291252 -1.129937 2.414133

H 2.706563 -0.498491 3.090179

H 3.805508 -1.902857 2.996044

H 4.016140 -0.503683 1.889175

C -0.972964 5.257877 0.824888

C -0.629773 4.535056 -0.314885

C -0.014147 3.270874 -0.221312

C 0.270407 2.771878 1.065361

C -0.074528 3.494745 2.206241

C -0.701276 4.738239 2.093305

H -1.451324 6.228618 0.724236

H -0.847256 4.944257 -1.298876

H 0.804622 1.829567 1.159302

H 0.164511 3.094378 3.188520

H -0.962372 5.303256 2.984120

C 0.292650 2.537940 -1.454914

H 0.326603 3.162911 -2.350878

C 0.492435 1.218239 -1.632644

H 0.653944 0.849535 -2.644379

Br 3.532687 0.463621 -0.891992

C -3.192504 -1.045098 -1.586006

C -1.799513 -0.439397 -2.002155

C -1.242076 -2.258912 -0.757982

C -2.812081 -2.293940 -0.748184

H -3.764581 -1.277623 -2.489432

H -3.171544 -3.236022 -1.174716

C -1.010768 -1.755533 -2.203185

H 0.044635 -1.594782 -2.440909

H -1.459360 -2.407623 -2.960240

C -1.173842 0.101441 -0.721612

H -1.517542 1.024293 -0.270530

C -0.831769 -1.034335 0.063918

H -0.962704 -1.031626 1.142823

H -1.855771 0.260113 -2.835242

H -0.781743 -3.207940 -0.480710

C -4.059455 -0.194608 -0.665555

C -3.473574 -2.077313 0.605867

O -4.573394 0.877190 -0.910501

O -3.420108 -2.793616 1.586161

N -4.172900 -0.870815 0.554759

C -4.912773 -0.344161 1.691238

H -5.596021 -1.107504 2.071427

H -4.227067 -0.056766 2.494365

H -5.466721 0.530372 1.347979

**8**

SCF done: -1168.210110

thermal Free Energies -1167.829296

Pd 1.106010 -0.432802 -0.312627

P 1.505560 -2.028054 1.325164

C 0.180510 -2.345748 2.589988

H -0.783550 -2.570362 2.127822

H 0.468766 -3.175679 3.244443

H 0.057064 -1.444770 3.198739

C 1.903060 -3.703453 0.636867

H 2.750386 -3.595284 -0.045625

H 2.156463 -4.407539 1.436944

H 1.047332 -4.088547 0.074538

C 2.971389 -1.636616 2.389199

H 2.783869 -0.704488 2.929671

H 3.152771 -2.445053 3.106403

H 3.838953 -1.492516 1.741747

C 2.018192 4.649296 0.797901

C 1.957580 3.663017 -0.182956

C 0.879593 2.757359 -0.247831

C -0.132785 2.869637 0.726635

C -0.071433 3.857241 1.707053

C 1.000266 4.752865 1.747446

H 2.861648 5.333620 0.822033

H 2.757259 3.581522 -0.913831

H -0.964781 2.178047 0.734638

H -0.864807 3.925572 2.446577

H 1.042936 5.520003 2.515714

C 0.907975 1.787687 -1.358628

H 1.811477 1.864929 -1.958618

C -0.026446 0.910156 -1.853105

H 0.230141 0.442352 -2.802521

Br 3.629663 -0.393812 -0.959876

C -3.590509 -0.662435 -1.492522

C -2.240479 -0.322844 -2.195859

C -1.636156 -1.808732 -0.585111

C -3.183970 -1.692097 -0.402698

H -4.298287 -1.063145 -2.225431

H -3.657258 -2.672348 -0.528178

C -1.552332 -1.708010 -2.126111

H -0.524167 -1.714467 -2.499524

H -2.119297 -2.491767 -2.641529

C -1.363758 0.522603 -1.241217

H -1.903223 1.408704 -0.891890

C -0.975426 -0.495694 -0.123137

H -1.244937 -0.193021 0.891073

H -2.368223 0.117543 -3.188171

H -1.228088 -2.719179 -0.142548

C -4.278213 0.465145 -0.733219

C -3.655534 -1.089267 0.909661

O -4.751273 1.488820 -1.183519

O -3.523061 -1.541578 2.031649

N -4.279691 0.125601 0.626941

C -4.843799 0.977938 1.661564

H -5.625702 0.441129 2.205875

H -4.067306 1.274500 2.372830

H -5.261354 1.857956 1.170731

**7’**

SCF done: -1168.158923

thermal Free Energies -1167.783664

Pd 0.787374 -0.657943 -0.507589

P 0.994727 -2.781811 0.786881

C -0.374097 -3.528002 1.816450

H -1.279471 -3.666156 1.217976

H -0.064432 -4.501355 2.212032

H -0.614296 -2.871768 2.657631

C 1.426272 -4.205885 -0.326935

H 2.289164 -3.921026 -0.932674

H 1.655258 -5.104070 0.257007

H 0.583433 -4.415411 -0.993144

C 2.392365 -2.750182 2.009959

H 2.163203 -2.031861 2.803360

H 2.549075 -3.739620 2.452958

H 3.296718 -2.423879 1.491628

C 1.973533 5.015497 0.872986

C 1.619128 4.306022 -0.273648

C 1.796977 2.911811 -0.354718

C 2.381277 2.257793 0.746119

C 2.740249 2.968400 1.891611

C 2.532118 4.348236 1.965787

H 1.819744 6.091125 0.910855

H 1.192217 4.834503 -1.123671

H 2.589388 1.194933 0.674770

H 3.202706 2.443842 2.724531

H 2.817852 4.899635 2.857760

C 1.404524 2.209382 -1.590651

H 1.438332 2.840846 -2.484212

C 1.014146 0.941760 -1.780867

H 0.798500 0.610640 -2.797479

Br 3.190143 -1.098813 -1.164971

C -3.262690 0.372613 1.379565

C -1.784161 -0.157743 1.501087

C -1.580948 1.612882 0.093497

C -3.124393 1.584787 0.420804

H -3.644031 0.636118 2.371028

H -3.416765 2.538512 0.870367

C -1.035232 1.192998 1.478472

H 0.052252 1.092543 1.491881

H -1.347739 1.869106 2.281476

C -1.435454 -0.721215 0.127003

H -1.727261 -1.717468 -0.185832

C -1.318981 0.361453 -0.729920

H -1.482525 0.324152 -1.800115

H -1.624827 -0.814955 2.355180

H -1.224995 2.549075 -0.331202

C -4.252877 -0.561437 0.697791

C -4.061269 1.281859 -0.741147

O -4.627787 -1.655283 1.072783

O -4.264844 1.957245 -1.728874

N -4.672716 0.047649 -0.484506

C -5.618465 -0.578194 -1.395424

H -5.814103 0.128659 -2.202626

H -5.200863 -1.504461 -1.801673

H -6.543723 -0.816925 -0.864581

**TS78’**

SCF done: -1168.152935

thermal Free Energies -1167.776125

Pd 0.984969 -0.646676 -0.338178

P 1.124225 -2.742338 0.640464

C -0.352091 -3.480158 1.498254

H -1.258588 -3.388389 0.894552

H -0.164956 -4.538474 1.710068

H -0.523682 -2.961538 2.445496

C 1.566658 -4.024568 -0.622088

H 2.474316 -3.698294 -1.135335

H 1.729353 -4.997555 -0.145431

H 0.757184 -4.109617 -1.353037

C 2.450003 -2.897489 1.926502

H 2.209355 -2.249093 2.774162

H 2.530592 -3.933848 2.273231

H 3.394151 -2.566771 1.488190

C 0.845291 5.023907 0.508468

C 0.554257 3.964326 -0.352367

C 1.348299 2.805790 -0.357353

C 2.469587 2.751545 0.490182

C 2.750632 3.807279 1.356365

C 1.937803 4.944152 1.373218

H 0.222135 5.914214 0.495246

H -0.273831 4.046762 -1.049893

H 3.108405 1.872839 0.464735

H 3.613362 3.744747 2.014257

H 2.163888 5.768039 2.044821

C 1.094473 1.714691 -1.332124

H 1.933911 1.513382 -1.996636

C -0.051056 1.028131 -1.629280

H 0.005415 0.430960 -2.541204

Br 3.540348 -0.784861 -0.891417

C -3.089024 0.006787 1.379496

C -1.530392 -0.110017 1.360706

C -1.921270 1.786444 0.166205

C -3.353977 1.308548 0.574477

H -3.454789 0.049781 2.411648

H -3.842002 2.091743 1.163366

C -1.152773 1.384549 1.447826

H -0.077284 1.560886 1.394114

H -1.546067 1.876590 2.345086

C -1.077513 -0.479605 -0.061190

H -1.541375 -1.384486 -0.459167

C -1.376111 0.804626 -0.901082

H -2.129633 0.636259 -1.678894

H -1.144941 -0.751374 2.154012

H -1.895222 2.838180 -0.115302

C -3.856221 -1.078174 0.639544

C -4.297926 0.897017 -0.546480

O -3.897255 -2.270233 0.882953

O -4.778701 1.598374 -1.413783

N -4.534105 -0.478880 -0.421852

C -5.372490 -1.231071 -1.341790

H -5.791504 -0.523323 -2.058218

H -4.779576 -1.988363 -1.863328

H -6.173325 -1.734202 -0.793060

**8’**

SCF done: -1168.201485

thermal Free Energies -1167.820533

Pd 0.984969 -0.646676 -0.338178

P 1.124225 -2.742338 0.640464

C -0.352091 -3.480158 1.498254

H -1.258588 -3.388389 0.894552

H -0.164956 -4.538474 1.710068

H -0.523682 -2.961538 2.445496

C 1.566658 -4.024568 -0.622088

H 2.474316 -3.698294 -1.135335

H 1.729353 -4.997555 -0.145431

H 0.757184 -4.109617 -1.353037

C 2.450003 -2.897489 1.926502

H 2.209355 -2.249093 2.774162

H 2.530592 -3.933848 2.273231

H 3.394151 -2.566771 1.488190

C 0.845291 5.023907 0.508468

C 0.554257 3.964326 -0.352367

C 1.348299 2.805790 -0.357353

C 2.469587 2.751545 0.490182

C 2.750632 3.807279 1.356365

C 1.937803 4.944152 1.373218

H 0.222135 5.914214 0.495246

H -0.273831 4.046762 -1.049893

H 3.108405 1.872839 0.464735

H 3.613362 3.744747 2.014257

H 2.163888 5.768039 2.044821

C 1.094473 1.714691 -1.332124

H 1.933911 1.513382 -1.996636

C -0.051056 1.028131 -1.629280

H 0.005415 0.430960 -2.541204

Br 3.540348 -0.784861 -0.891417

C -3.089024 0.006787 1.379496

C -1.530392 -0.110017 1.360706

C -1.921270 1.786444 0.166205

C -3.353977 1.308548 0.574477

H -3.454789 0.049781 2.411648

H -3.842002 2.091743 1.163366

C -1.152773 1.384549 1.447826

H -0.077284 1.560886 1.394114

H -1.546067 1.876590 2.345086

C -1.077513 -0.479605 -0.061190

H -1.541375 -1.384486 -0.459167

C -1.376111 0.804626 -0.901082

H -2.129633 0.636259 -1.678894

H -1.144941 -0.751374 2.154012

H -1.895222 2.838180 -0.115302

C -3.856221 -1.078174 0.639544

C -4.297926 0.897017 -0.546480

O -3.897255 -2.270233 0.882953

O -4.778701 1.598374 -1.413783

N -4.534105 -0.478880 -0.421852

C -5.372490 -1.231071 -1.341790

H -5.791504 -0.523323 -2.058218

H -4.779576 -1.988363 -1.863328

H -6.173325 -1.734202 -0.793060

**9’**

SCF done: -1154.783996

thermal Free Energies -1154.400872

Pd 1.358837 -0.783119 -0.710645

P 1.674080 -2.611762 0.746606

C 0.749769 -2.511530 2.349225

H -0.325515 -2.398788 2.179580

H 0.926023 -3.413984 2.943601

H 1.102733 -1.641635 2.911043

C 1.265516 -4.285319 0.061699

H 1.851349 -4.464211 -0.844256

H 1.495875 -5.061615 0.798806

H 0.203983 -4.343125 -0.193167

C 3.453214 -2.765553 1.252265

H 3.775535 -1.847899 1.752086

H 3.580749 -3.611506 1.936080

H 4.079834 -2.924594 0.369727

C 3.553190 3.971554 0.374023

C 3.125307 3.104543 -0.627034

C 1.908946 2.396838 -0.512936

C 1.141672 2.581608 0.658464

C 1.571706 3.448894 1.657479

C 2.775806 4.147513 1.520324

H 4.488632 4.510319 0.258662

H 3.730040 2.979967 -1.521800

H 0.212049 2.045580 0.800390

H 0.966074 3.582322 2.548913

H 3.104616 4.823132 2.304254

C 1.544201 1.546579 -1.652920

H 2.312689 1.538834 -2.426396

C 0.400096 0.863595 -2.002598

H 0.385852 0.451654 -3.010327

C -3.344151 0.084543 -1.141786

C -2.078878 0.183775 -2.047262

C -1.593329 -1.557776 -0.663825

C -3.013911 -1.094763 -0.191126

H -4.227159 -0.091092 -1.763412

H -3.712436 -1.934434 -0.264869

C -1.731829 -1.319112 -2.185970

H -0.811143 -1.519386 -2.742833

H -2.541635 -1.902567 -2.634612

C -0.892849 0.715384 -1.199777

H -1.136248 1.655801 -0.696289

C -0.620844 -0.461098 -0.236172

H -0.573270 -0.243981 0.831499

H -2.240517 0.744407 -2.970137

H -1.345704 -2.566562 -0.333086

C -3.632422 1.263605 -0.217351

C -3.091123 -0.510826 1.214022

O -3.896489 2.402670 -0.532263

O -2.798943 -1.065439 2.257152

N -3.511787 0.809973 1.107223

C -3.727528 1.668018 2.266805

H -4.592144 1.318080 2.837165

H -2.848545 1.645533 2.916112

H -3.908796 2.679170 1.900863

**9**

SCF done: -1742.406985

thermal Free Energies -1742.015968

Pd 2.551851 -0.033767 0.027771

P 4.534440 -1.547765 0.472107

C 4.227349 -2.849015 1.766261

H 3.342658 -3.431319 1.499943

H 5.094472 -3.513024 1.861591

H 4.037943 -2.360534 2.726803

C 5.027294 -2.562116 -1.006620

H 5.366381 -1.901374 -1.811898

H 5.824847 -3.275217 -0.767032

H 4.120742 -3.080168 -1.328793

C 6.188532 -0.873955 1.037717

H 6.066268 -0.337399 1.984451

H 6.923406 -1.674994 1.179182

H 6.579927 -0.168324 0.297110

C 4.036547 3.751381 2.538984

C 4.008566 2.663560 1.666488

C 3.810830 2.850217 0.289401

C 3.657022 4.161355 -0.190042

C 3.682462 5.251103 0.682568

C 3.871095 5.050072 2.050784

H 4.184474 3.583940 3.602886

H 4.115769 1.652512 2.049290

H 3.529741 4.328192 -1.256262

H 3.561408 6.257214 0.289216

H 3.892685 5.897447 2.731014

C 3.851185 1.703102 -0.670357

H 4.841586 1.276733 -0.826817

C 2.888022 1.403286 -1.622373

H 3.165055 0.759719 -2.456739

C -0.821414 2.209619 -2.592123

C 0.569874 1.529122 -2.756923

C -0.321274 0.449313 -0.956833

C -1.429854 1.496286 -1.348210

H -1.413695 2.072044 -3.503455

H -2.367034 0.924502 -1.511895

C 0.183527 0.081823 -2.373281

H 1.017328 -0.625908 -2.337809

H -0.613730 -0.313024 -3.015322

C 1.475185 1.965534 -1.578372

H 1.488266 3.053490 -1.461018

C 0.891090 1.173312 -0.374990

H 0.716301 1.755312 0.534115

H 1.014086 1.685595 -3.744880

H -0.730392 -0.378501 -0.382628

C -0.797720 3.686445 -2.244898

C -1.705502 2.613988 -0.370417

O -0.423526 4.627435 -2.920064

O -2.231838 2.557332 0.735776

N -1.300333 3.822921 -0.947613

C -1.385624 5.109662 -0.280798

H -2.019009 5.794465 -0.853440

H -1.807962 4.940767 0.710749

H -0.390038 5.554573 -0.191870

O -3.897470 -0.329124 -1.076932

C -3.500270 -1.132631 -0.131443

O -2.583896 -2.021968 -0.366703

O -3.985741 -1.019459 1.076442

Cs -1.406534 -1.910009 2.376133

C 1.330355 -2.573900 0.316533

O 1.874342 -2.603441 -0.843403

O 0.701861 -3.541811 0.844030

O 1.392473 -1.426601 1.025443

Cs -0.824789 -3.680326 -1.878406

Cs -5.242026 1.529396 0.690540

**TS9-10**

SCF done: -1742.371106

thermal Free Energies -1742.006345

Pd 2.626700 0.176900 -0.059800

P 4.803900 -1.041200 0.372000

C 4.749500 -2.264000 1.771500

H 3.990000 -3.010400 1.531800

H 5.722000 -2.750600 1.910000

H 4.459400 -1.755300 2.695100

C 5.326400 -2.118200 -1.053800

H 5.528800 -1.499300 -1.934800

H 6.221900 -2.704400 -0.815900

H 4.478600 -2.774900 -1.261200

C 6.402200 -0.134700 0.747200

H 6.289000 0.455900 1.662200

H 7.240100 -0.829300 0.879400

H 6.645300 0.552600 -0.070400

C 3.791700 4.134900 2.309700

C 3.858000 3.051900 1.433700

C 3.497000 3.193300 0.084100

C 3.085200 4.458700 -0.365100

C 3.016500 5.543300 0.511300

C 3.367100 5.385200 1.852800

H 4.069300 4.001000 3.352300

H 4.167600 2.076100 1.795900

H 2.829700 4.596000 -1.412100

H 2.693100 6.512900 0.141200

H 3.314900 6.229100 2.535800

C 3.629600 2.059500 -0.882700

H 4.656100 1.762000 -1.094900

C 2.662000 1.629900 -1.775700

H 2.979500 1.019100 -2.619800

C -1.171700 1.836000 -2.581000

C 0.314600 1.451200 -2.828900

C -0.282400 0.131500 -1.067400

C -1.551600 0.997600 -1.335200

H -1.753700 1.585800 -3.479600

H -2.660900 0.097300 -1.396200

C 0.226800 -0.062800 -2.513800

H 1.187100 -0.588000 -2.554400

H -0.501100 -0.576600 -3.153300

C 1.181900 1.975800 -1.658700

H 1.029200 3.045400 -1.487000

C 0.786700 1.056200 -0.474200

H 0.505800 1.571400 0.447600

H 0.686400 1.730600 -3.820400

H -0.476600 -0.787800 -0.525300

C -1.502700 3.276000 -2.221100

C -1.962800 1.962600 -0.331800

O -1.400600 4.284800 -2.901900

O -2.295400 1.803600 0.858700

N -2.019800 3.252800 -0.926600

C -2.429000 4.448800 -0.221800

H -1.956600 5.306900 -0.704300

H -3.519000 4.593000 -0.261300

H -2.123200 4.373900 0.824400

O -3.669000 -0.560600 -1.189900

C -3.412700 -1.511900 -0.251000

O -2.433700 -2.300600 -0.438600

O -4.138600 -1.511500 0.798900

Cs -1.283100 -1.331000 2.304000

C 1.604500 -2.412800 0.676600

O 2.247800 -2.663800 -0.402400

O 0.875400 -3.254400 1.293000

O 1.645000 -1.165900 1.180000

Cs -0.307400 -4.040800 -1.396100

Cs -5.358900 1.133500 0.545700

**10**

SCF done: -1742.393252

thermal Free Energies -1742.011392

Pd 0.743805 1.370645 -0.885926

P 3.329545 0.622168 3.500432

C 2.212935 -0.816661 3.044708

H 1.714048 -0.602823 2.093226

H 2.781942 -1.751722 2.951757

H 1.447678 -0.939620 3.818748

C 4.578525 0.473621 2.099163

H 5.398110 1.181750 2.272378

H 4.985064 -0.544837 2.028545

H 4.071008 0.727233 1.161258

C 4.325891 -0.192248 4.872099

H 3.686758 -0.340254 5.749096

H 4.735086 -1.163885 4.566913

H 5.152283 0.462782 5.168853

C 1.271705 4.489317 2.665685

C 1.431397 3.889835 1.417961

C 0.577579 4.208463 0.348189

C -0.422183 5.171576 0.558238

C -0.580064 5.776483 1.806713

C 0.261693 5.434541 2.865898

H 1.929758 4.205095 3.482430

H 2.207483 3.143290 1.271696

H -1.070423 5.461082 -0.263504

H -1.363072 6.517058 1.947820

H 0.133308 5.900948 3.839224

C 0.796061 3.597700 -0.996080

H 1.776957 3.804625 -1.427643

C -0.169932 3.108907 -1.870896

H 0.100680 3.003439 -2.921460

C -3.890387 1.921004 -2.037797

C -2.480115 2.210859 -2.608356

C -2.138862 0.392091 -1.260893

C -3.596642 0.826945 -1.008055

H -4.541800 1.609939 -2.870826

H -3.598466 -5.782414 -0.955834

C -1.978520 0.749133 -2.758972

H -0.941952 0.666411 -3.106284

H -2.626846 0.153971 -3.412149

C -1.593578 2.753946 -1.459825

H -2.090808 3.571265 -0.930415

C -1.316423 1.500619 -0.596525

H -1.442931 1.612689 0.482140

H -2.467397 2.828383 -3.512979

H -1.839599 -0.624302 -0.986933

C -4.659206 2.994805 -1.270653

C -4.096396 1.275975 0.238574

O -5.121494 4.051784 -1.687513

O -4.018891 0.807692 1.399523

N -4.824201 2.507196 0.006234

C -5.555603 3.206959 1.037132

H -5.209672 4.242891 1.116851

H -6.631539 3.226546 0.818902

H -5.375613 2.678453 1.975620

O -3.608005 -5.070610 -0.294832

C -2.240443 -4.892373 0.035614

O -1.430986 -5.617030 -0.578600

O -2.043181 -3.994719 0.889781

Cs 0.879550 -3.621843 0.076136

C 2.470192 -0.591682 -0.731128

O 2.925171 0.596244 -1.038126

O 3.205078 -1.625365 -0.705142

O 1.186049 -0.657422 -0.435625

Cs 5.678650 -0.216901 -1.593883

Cs -4.363736 -2.089330 0.737831

**11**

SCF done: -1732.969058

thermal Free Energies -1732.411843

Pd 0.168142 -1.830772 0.332739

P 0.569155 -3.390640 -1.301276

C -0.477997 -4.890126 -0.923341

H -1.535356 -4.623191 -1.006960

H -0.255463 -5.715633 -1.610077

H -0.289014 -5.215342 0.103868

C 0.224971 -3.187614 -3.122545

H 0.920486 -2.468939 -3.563594

H 0.336210 -4.147508 -3.639619

H -0.794015 -2.817424 -3.265030

C 2.278747 -4.130418 -1.322133

H 2.512425 -4.510390 -0.323210

H 2.351766 -4.948673 -2.047899

H 3.007956 -3.354277 -1.566297

C 6.481450 0.912339 1.178494

C 5.832218 0.341980 0.085864

C 4.451390 0.072392 0.119266

C 3.750901 0.358325 1.305092

C 4.401300 0.925712 2.400981

C 5.766474 1.211532 2.340987

H 7.546476 1.122186 1.124200

H 6.394521 0.112517 -0.816453

H 2.699302 0.099438 1.378368

H 3.841157 1.135023 3.308810

H 6.271521 1.652997 3.195831

C 3.811613 -0.531237 -1.064565

H 4.450887 -1.221992 -1.616516

C 2.580488 -0.318079 -1.563559

H 2.310992 -0.882882 -2.455535

C 0.242476 2.717684 -1.874689

C 0.986179 1.460084 -2.376398

C -0.881135 0.591089 -1.345553

C -0.855316 2.092604 -1.031480

H -0.126936 3.275653 -2.749190

H -2.666353 2.477007 -1.535282

C -0.234248 0.576290 -2.748443

H 0.038631 -0.428411 -3.081291

H -0.870981 1.038435 -3.510622

C 1.570252 0.716155 -1.133032

H 2.079579 1.422902 -0.468429

C 0.277246 0.202613 -0.445840

H 0.174296 0.485873 0.603030

H 1.718898 1.638189 -3.167789

H -1.821036 0.055645 -1.215956

C 0.906914 3.740314 -0.955491

C -0.818496 2.704392 0.272788

O 1.839025 4.490900 -1.192602

O -1.455800 2.473660 1.311060

N 0.174792 3.735053 0.222964

C 0.451608 4.630849 1.327632

H 0.336641 5.674072 1.014684

H -0.257356 4.394985 2.123102

H 1.476402 4.493875 1.688454

O -3.651342 2.542643 -1.697304

C -4.148563 3.623217 -0.911658

H -5.241171 3.592331 -0.962165

H -3.833932 3.545190 0.136237

H -3.813362 4.590292 -1.311538

C -5.470152 -0.340211 -1.997945

H -6.389325 0.248103 -1.859133

H -5.144642 -0.234768 -3.041794

H -5.703567 -1.392548 -1.811425

O -4.443900 0.042179 -1.094716

H -4.186097 0.984065 -1.288239

C -4.489533 0.940851 2.156429

H -4.469393 0.812370 3.243131

H -3.538483 1.380427 1.831415

H -5.310253 1.629949 1.911550

C -3.502146 -2.702254 3.628492

H -2.771741 -3.409774 4.031888

H -3.741686 -1.964926 4.409070

H -4.418711 -3.252956 3.378506

O -2.933600 -2.100498 2.475088

H -3.593433 -1.452862 2.093222

O -4.718323 -0.345614 1.577860

H -4.653578 -0.245723 0.593274

C -0.209999 -0.092484 3.194266

H 0.870207 0.049818 3.289024

H -0.655340 0.785891 2.709917

H -0.632198 -0.207063 4.200393

O -0.422584 -1.288294 2.438378

H -1.387950 -1.548043 2.494652

**TS11-12**

SCF done: -1732.960278

thermal Free Energies -1732.358721

Pd 0.274500 -1.889700 0.373300

P 0.684000 -3.371600 -1.342800

C -0.230600 -4.919100 -0.840700

H -1.305600 -4.718300 -0.829500

H -0.021000 -5.741300 -1.534800

H 0.073800 -5.216600 0.167300

C 0.152600 -3.210200 -3.118600

H 0.735300 -2.439100 -3.627600

H 0.301800 -4.164700 -3.635700

H -0.905100 -2.937400 -3.165100

C 2.429800 -3.991400 -1.497700

H 2.772600 -4.334800 -0.517200

H 2.490800 -4.818500 -2.213900

H 3.079400 -3.174200 -1.817700

C 6.558200 1.141600 0.345100

C 5.803300 0.566900 -0.674800

C 4.458000 0.208400 -0.469900

C 3.907300 0.408300 0.809000

C 4.664600 0.979100 1.832300

C 5.989400 1.354900 1.603400

H 7.591500 1.422200 0.159200

H 6.251000 0.405300 -1.652700

H 2.892400 0.080100 1.010000

H 4.220800 1.121200 2.814400

H 6.577700 1.799600 2.401300

C 3.700800 -0.387600 -1.585900

H 4.302700 -0.995700 -2.263000

C 2.400000 -0.243000 -1.898900

H 2.047500 -0.772800 -2.783100

C -0.173100 2.626800 -1.786100

C 0.535200 1.387300 -2.382300

C -1.028300 0.455800 -1.000400

C -1.152600 2.000500 -0.778000

H -0.684700 3.167100 -2.593500

H -2.388000 2.216900 -1.127400

C -0.678200 0.434400 -2.503500

H -0.419200 -0.562800 -2.869700

H -1.484800 0.844900 -3.118000

C 1.395500 0.691900 -1.273300

H 1.961800 1.432600 -0.696700

C 0.294400 0.097800 -0.348200

H 0.397400 0.420800 0.691500

H 1.099800 1.586400 -3.297100

H -1.907400 -0.108300 -0.689600

C 0.633300 3.642800 -0.989500

C -0.820300 2.599100 0.530800

O 1.510500 4.395300 -1.374300

O -1.250300 2.335600 1.653000

N 0.150100 3.606200 0.316000

C 0.656500 4.472500 1.363900

H 0.553200 5.520700 1.067800

H 0.074400 4.273300 2.265000

H 1.716300 4.271800 1.552900

O -3.593600 2.279200 -1.618300

C -4.264100 3.380700 -1.074100

H -5.139400 3.082400 -0.468500

H -3.611200 3.977600 -0.409500

H -4.630200 4.063900 -1.860400

C -5.521900 -0.389600 -1.745800

H -6.439200 0.191200 -1.558600

H -5.257500 -0.270800 -2.806400

H -5.747200 -1.446800 -1.563700

O -4.453600 -0.002900 -0.904800

H -4.139400 0.944500 -1.180500

C -4.463400 0.964100 2.307900

H -4.498900 0.842100 3.395600

H -3.508700 1.429500 2.033200

H -5.285700 1.629600 2.012100

C -3.347700 -2.707900 3.619600

H -2.629600 -3.462300 3.955600

H -3.540400 -2.014000 4.451000

H -4.288600 -3.211200 3.362900

O -2.796800 -2.050600 2.487400

H -3.468200 -1.376500 2.153400

O -4.625100 -0.328200 1.723400

H -4.605600 -0.218800 0.727600

C 0.068600 -0.289200 3.282700

H 1.159400 -0.215500 3.297000

H -0.364600 0.644800 2.907700

H -0.281100 -0.471600 4.305900

O -0.279400 -1.403400 2.451600

H -1.271400 -1.581400 2.516300

10(B3LYP-D3)

SCF done: -1741.344183

thermal Free Energies -1740.952113

Pd 2.686788 0.153120 -0.179802

P 4.878675 -0.858628 0.479125

C 4.804659 -1.394174 2.249317

H 3.864225 -1.938199 2.391370

H 5.664173 -2.021889 2.520123

H 4.782207 -0.506880 2.894928

C 5.254045 -2.449482 -0.383267

H 5.522810 -2.242468 -1.426458

H 6.076893 -2.987879 0.106790

H 4.321068 -3.028015 -0.377949

C 6.539825 -0.014197 0.432540

H 6.507862 0.918231 1.010505

H 7.324557 -0.662623 0.845519

H 6.800662 0.235083 -0.603877

C 3.681096 4.077211 2.129734

C 3.797957 3.011840 1.245826

C 3.392353 3.138269 -0.087260

C 2.880987 4.368306 -0.513308

C 2.759681 5.435199 0.371570

C 3.157832 5.292956 1.696387

H 3.996953 3.956496 3.165165

H 4.181183 2.050215 1.590207

H 2.587567 4.490354 -1.555977

H 2.357147 6.383658 0.018683

H 3.065119 6.127388 2.389915

C 3.563798 2.024898 -1.056423

H 4.600366 1.774106 -1.300392

C 2.587641 1.546603 -1.911757

H 2.906948 0.933018 -2.758657

C -1.220387 1.565542 -2.642120

C 0.260973 1.254139 -2.916442

C -0.235881 -0.087408 -1.154063

C -1.496326 0.748252 -1.384416

H -1.810564 1.265683 -3.527463

H -3.131950 -0.631788 -1.445771

C 0.260986 -0.254715 -2.595988

H 1.247369 -0.735852 -2.651662

H -0.454909 -0.804296 -3.227882

C 1.105014 1.824596 -1.769136

H 0.890435 2.889399 -1.597011

C 0.786183 0.894759 -0.589673

H 0.505212 1.394792 0.346443

H 0.613673 1.544717 -3.915489

H -0.359589 -1.014118 -0.587252

C -1.649804 2.976257 -2.299624

C -1.981801 1.627501 -0.405582

O -1.579286 3.990581 -2.970397

O -2.211897 1.497869 0.820320

N -2.223205 2.906180 -1.042028

C -2.663901 4.078162 -0.338425

H -3.638424 4.439828 -0.704345

H -2.728545 3.821273 0.725753

H -1.946199 4.896444 -0.474674

O -3.971312 -0.921391 -1.009856

C -3.563538 -1.682191 0.091296

O -2.536479 -2.375409 -0.073471

O -4.259558 -1.535718 1.115102

Cs -1.070753 -1.093922 2.313892

C 1.614811 -2.379446 0.605698

O 2.167682 -2.618258 -0.517530

O 0.844956 -3.183327 1.211586

O 1.796032 -1.181759 1.169486

Cs -0.415087 -3.930756 -1.377066

Cs -5.219152 1.241914 0.625292

TS11-12(B3LYP-D3)

SCF done: -1731.906782

thermal Free Energies -1731.511212

Pd -0.485447 -1.378429 0.430869

P -0.915917 -2.722077 -1.441905

C -2.367187 -3.741947 -0.911144

H -3.235970 -3.092340 -0.768554

H -2.600915 -4.512026 -1.653206

H -2.130430 -4.217082 0.043860

C -1.383593 -2.357880 -3.196059

H -0.562173 -1.876661 -3.730459

H -1.628029 -3.300696 -3.695860

H -2.255302 -1.699701 -3.224337

C 0.383396 -4.016593 -1.665689

H 0.617615 -4.455646 -0.692188

H 0.039826 -4.800670 -2.347957

H 1.291040 -3.553074 -2.057131

C 6.054431 -1.071158 0.358560

C 5.213049 -1.234978 -0.735712

C 3.821890 -1.187371 -0.583746

C 3.293876 -1.009062 0.701470

C 4.136441 -0.856306 1.799214

C 5.518257 -0.878033 1.630430

H 7.130812 -1.097128 0.219814

H 5.634075 -1.386546 -1.726262

H 2.216119 -1.036630 0.845230

H 3.709861 -0.736535 2.791010

H 6.175496 -0.758013 2.486007

C 2.948365 -1.365344 -1.761886

H 3.257536 -2.141402 -2.462714

C 1.853365 -0.652916 -2.054731

H 1.305729 -0.917512 -2.961178

C 0.476101 2.937042 -1.670024

C 0.670602 1.552658 -2.322220

C -0.935458 1.167678 -0.769391

C -0.567593 2.662825 -0.573959

H 0.147046 3.654594 -2.431658

H -1.893479 3.153679 -0.717529

C -0.787766 1.055143 -2.295286

H -0.904834 0.034051 -2.665408

H -1.475692 1.718560 -2.828885

C 1.356025 0.568700 -1.328887

H 2.223336 1.031231 -0.838943

C 0.244513 0.357803 -0.269415

H 0.584374 0.651025 0.732088

H 1.166663 1.581473 -3.295851

H -1.901711 0.898184 -0.341154

C 1.669735 3.535590 -0.949247

C 0.086080 3.027990 0.687393

O 2.726895 3.910590 -1.409130

O -0.287761 2.868601 1.843635

N 1.337350 3.603002 0.394015

C 2.248141 4.101954 1.397862

H 3.038324 3.372309 1.604404

H 2.711168 5.022880 1.037767

H 1.674855 4.286356 2.307012

O -3.117753 3.268580 -0.775077

C -3.479585 4.164671 0.242369

H -4.356845 3.795438 0.794638

H -2.661818 4.295669 0.969614

H -3.734619 5.154105 -0.163403

C -4.224272 0.470209 -1.659589

H -4.119344 1.202376 -2.470144

H -3.382525 -0.243313 -1.738976

H -5.157343 -0.084965 -1.810863

O -4.243465 1.112140 -0.411975

H -3.737803 2.038966 -0.532338

C -2.837421 0.725603 2.426776

H -2.337601 0.229958 3.267509

H -2.143441 1.454422 1.988214

H -3.715676 1.253572 2.816574

C -2.638286 -3.582580 2.652355

H -1.895797 -4.369978 2.800400

H -3.109558 -3.358879 3.616164

H -3.408691 -3.947428 1.962765

O -1.968867 -2.447342 2.135688

H -2.621416 -1.730688 1.890069

O -3.216503 -0.279584 1.499875

H -3.669211 0.189227 0.725430

C 0.679382 0.215280 3.240815

H 1.585564 0.545084 2.725890

H -0.059257 1.017351 3.172223

H 0.928849 -0.003293 4.283932

O 0.243419 -0.974132 2.583922

H -0.547641 -1.356042 3.005763

10(TPSS)

SCF done: -1742.593895

thermal Free Energies -1742.215612

Pd 2.665962 0.519408 -0.219292

P 5.042280 -0.206503 0.106057

C 5.335709 -1.166199 1.686081

H 4.637477 -2.008824 1.721786

H 6.370912 -1.532277 1.741095

H 5.123619 -0.516843 2.543042

C 5.576687 -1.454641 -1.184426

H 5.635464 -0.966126 -2.165582

H 6.548071 -1.909760 -0.944650

H 4.791990 -2.218949 -1.216234

C 6.517756 0.962728 0.122021

H 6.401649 1.679932 0.944928

H 7.467347 0.424044 0.250011

H 6.552440 1.524676 -0.820730

C 1.606777 4.072453 2.782380

C 2.224835 3.197541 1.879096

C 2.286882 3.503534 0.504052

C 1.710341 4.713243 0.063411

C 1.083529 5.584650 0.964679

C 1.030560 5.268704 2.329054

H 1.572684 3.817619 3.842332

H 2.646483 2.253110 2.223598

H 1.761901 4.973053 -0.994090

H 0.642277 6.512324 0.599802

H 0.548049 5.947896 3.031919

C 3.009359 2.609612 -0.465429

H 4.092360 2.771478 -0.475811

C 2.444902 2.082459 -1.670989

H 3.067797 1.937475 -2.557059

C -1.232615 1.378799 -2.902092

C 0.321841 1.318089 -3.010060

C -0.143478 -0.123438 -1.292901

C -1.491157 0.548186 -1.634134

H -1.659327 0.950817 -3.826304

H -2.804967 -1.077817 -1.506283

C 0.527376 -0.187353 -2.688160

H 1.584158 -0.482018 -2.627352

H -0.010854 -0.836104 -3.397613

C 0.920322 2.015754 -1.762976

H 0.454264 3.001069 -1.614073

C 0.667141 0.993164 -0.622663

H 0.246216 1.410347 0.298427

H 0.723046 1.675536 -3.967094

H -0.204889 -1.063954 -0.743608

C -1.942733 2.711400 -2.664716

C -2.184932 1.381235 -0.718241

O -2.043705 3.690337 -3.409826

O -2.507824 1.248939 0.505097

N -2.564833 2.593882 -1.422434

C -3.297540 3.685717 -0.810248

H -4.389608 3.577590 -0.943635

H -3.067954 3.709943 0.261212

H -2.990870 4.615434 -1.301531

O -3.601421 -1.539834 -1.096760

C -3.136109 -2.233630 0.053006

O -1.964803 -2.713048 -0.009621

O -3.950688 -2.259259 1.016601

Cs -0.851398 -0.979506 2.329870

C 2.067333 -1.804907 0.978409

O 2.411207 -1.739930 -0.309755

O 1.623714 -2.880754 1.496148

O 2.158872 -0.674934 1.651126

Cs 0.496305 -3.976917 -1.076725

Cs -5.398977 0.292481 0.414894

TS11-12(TPSS)

SCF done: -1733.152981

thermal Free Energies -1732.562824

Pd 0.487134 -2.032495 0.506785

P 1.559751 -3.377905 -0.991817

C 0.970038 -5.109265 -0.551172

H -0.113036 -5.176989 -0.707059

H 1.475871 -5.862143 -1.172340

H 1.185252 -5.310622 0.505246

C 1.260370 -3.399281 -2.841343

H 1.693251 -2.513081 -3.317123

H 1.713728 -4.298522 -3.279919

H 0.180649 -3.407050 -3.030012

C 3.412630 -3.608657 -0.878138

H 3.670252 -3.854018 0.159064

H 3.744561 -4.420460 -1.539875

H 3.916413 -2.675572 -1.146740

C 6.200204 2.288377 0.971691

C 5.684917 1.488333 -0.052399

C 4.404830 0.898460 0.055106

C 3.677577 1.103829 1.249463

C 4.195448 1.902086 2.276531

C 5.454048 2.504293 2.139479

H 7.183958 2.742921 0.859902

H 6.267947 1.328390 -0.959912

H 2.721860 0.600925 1.386209

H 3.619461 2.042982 3.190744

H 5.855210 3.124507 2.939930

C 3.902967 0.076525 -1.061289

H 4.682479 -0.442648 -1.627008

C 2.629594 -0.072770 -1.497804

H 2.474119 -0.728409 -2.357489

C -0.334589 2.345275 -1.820403

C 0.554728 1.160586 -2.277369

C -0.971667 0.140653 -0.907033

C -1.265136 1.675329 -0.789820

H -0.881905 2.738437 -2.689903

H -2.485251 1.790018 -1.206779

C -0.529557 0.056445 -2.385739

H -0.130220 -0.931856 -2.649068

H -1.340076 0.326203 -3.074506

C 1.412924 0.697337 -1.049331

H 1.778428 1.570550 -0.487513

C 0.350200 -0.022913 -0.170200

H 0.355621 0.319881 0.873968

H 1.151001 1.360652 -3.174801

H -1.793830 -0.503939 -0.586827

C 0.311220 3.519718 -1.091358

C -1.033065 2.392591 0.490048

O 1.084290 4.369955 -1.523165

O -1.460512 2.161801 1.630096

N -0.184365 3.499699 0.217040

C 0.194635 4.485163 1.222680

H -0.009508 5.494119 0.846422

H -0.399003 4.274921 2.117254

H 1.264949 4.404126 1.450973

O -3.632317 1.867645 -1.861168

C -3.981725 3.231394 -1.996340

H -3.825336 3.590891 -3.031271

H -5.043903 3.390968 -1.739786

H -3.385450 3.883969 -1.327488

C -6.070017 -0.269864 -1.569274

H -6.315330 0.176268 -2.545653

H -5.342955 -1.085266 -1.735192

H -6.984245 -0.700867 -1.140814

O -5.584739 0.719634 -0.665849

H -4.757403 1.172951 -1.112646

C -4.674474 0.879222 2.553563

H -4.486175 0.479344 3.557979

H -3.785227 1.427700 2.211070

H -5.532861 1.565040 2.604629

C -3.733363 -3.230389 1.684452

H -3.011163 -4.032891 1.493755

H -4.030333 -3.260433 2.744907

H -4.624597 -3.397974 1.063123

O -3.106931 -1.989747 1.336977

H -3.800188 -1.242176 1.485737

O -4.982202 -0.236497 1.700965

H -5.222853 0.135350 0.778658

C -0.603855 -0.639572 3.254658

H 0.429920 -0.647958 3.614620

H -0.852883 0.336104 2.815450

H -1.279927 -0.850291 4.094611

O -0.724244 -1.700240 2.279119

H -1.705169 -1.766463 1.963600

10(M06-2X)

SCF done: -1741.483473

thermal Free Energies -1741.084315

Pd 2.621774 0.258626 -0.050208

P 4.832138 -0.945388 0.516272

C 4.602595 -2.218116 1.834956

H 3.879672 -2.945606 1.459776

H 5.549636 -2.715306 2.069885

H 4.187365 -1.751586 2.730792

C 5.386754 -1.994227 -0.903708

H 5.674789 -1.362128 -1.749395

H 6.230433 -2.636312 -0.629614

H 4.519155 -2.598436 -1.181279

C 6.436796 -0.157439 1.042603

H 6.297704 0.351371 2.001463

H 7.231553 -0.902499 1.154260

H 6.751269 0.586925 0.304210

C 3.882516 4.181273 2.124010

C 3.995798 3.146556 1.203396

C 3.399999 3.245231 -0.060077

C 2.713337 4.417814 -0.389101

C 2.598077 5.454404 0.533704

C 3.177780 5.338151 1.793607

H 4.343971 4.084847 3.102156

H 4.534075 2.237363 1.461477

H 2.286870 4.527255 -1.381705

H 2.060996 6.357653 0.260905

H 3.089137 6.146876 2.512379

C 3.573274 2.151351 -1.054205

H 4.607033 1.866850 -1.252297

C 2.616105 1.639143 -1.886101

H 2.952248 0.981542 -2.688080

C -1.168672 1.450372 -2.726134

C 0.340966 1.205021 -2.923875

C -0.172720 -0.106284 -1.133090

C -1.459699 0.660962 -1.450543

H -1.703534 1.100866 -3.622297

H -3.112662 -0.573382 -1.473869

C 0.403160 -0.296971 -2.549737

H 1.411009 -0.726630 -2.535778

H -0.250774 -0.895660 -3.196820

C 1.113567 1.849381 -1.758412

H 0.847121 2.901732 -1.626486

C 0.788488 0.942236 -0.549355

H 0.404212 1.462822 0.332972

H 0.721984 1.480703 -3.911930

H -0.277585 -1.015328 -0.542162

C -1.682136 2.854071 -2.442171

C -2.019380 1.551258 -0.525481

O -1.648023 3.845509 -3.147226

O -2.284926 1.450282 0.698982

N -2.288503 2.794835 -1.201271

C -2.839844 3.956179 -0.555521

H -3.905389 4.095195 -0.791945

H -2.713278 3.839250 0.523610

H -2.309228 4.844651 -0.906031

O -3.942686 -0.880440 -1.023078

C -3.503689 -1.688017 0.024100

O -2.480684 -2.372820 -0.196348

O -4.166219 -1.595377 1.079824

Cs -1.155078 -1.079738 2.237570

C 1.603144 -2.256708 0.716241

O 2.230891 -2.462282 -0.382796

O 0.842087 -3.110888 1.262488

O 1.706073 -1.062084 1.291383

Cs -0.238363 -3.837896 -1.382127

Cs -5.245493 1.127236 0.702624

TS11-12(M06-2X)

SCF done: -1732.050073

thermal Free Energies -1731.494756

Pd -0.485447 -1.378429 0.430869

P -0.915917 -2.722077 -1.441905

C -2.367187 -3.741947 -0.911144

H -3.235970 -3.092340 -0.768554

H -2.600915 -4.512026 -1.653206

H -2.130430 -4.217082 0.043860

C -1.383593 -2.357880 -3.196059

H -0.562173 -1.876661 -3.730459

H -1.628029 -3.300696 -3.695860

H -2.255302 -1.699701 -3.224337

C 0.383396 -4.016593 -1.665689

H 0.617615 -4.455646 -0.692188

H 0.039826 -4.800670 -2.347957

H 1.291040 -3.553074 -2.057131

C 6.054431 -1.071158 0.358560

C 5.213049 -1.234978 -0.735712

C 3.821890 -1.187371 -0.583746

C 3.293876 -1.009062 0.701470

C 4.136441 -0.856306 1.799214

C 5.518257 -0.878033 1.630430

H 7.130812 -1.097128 0.219814

H 5.634075 -1.386546 -1.726262

H 2.216119 -1.036630 0.845230

H 3.709861 -0.736535 2.791010

H 6.175496 -0.758013 2.486007

C 2.948365 -1.365344 -1.761886

H 3.257536 -2.141402 -2.462714

C 1.853365 -0.652916 -2.054731

H 1.305729 -0.917512 -2.961178

C 0.476101 2.937042 -1.670024

C 0.670602 1.552658 -2.322220

C -0.935458 1.167678 -0.769391

C -0.567593 2.662825 -0.573959

H 0.147046 3.654594 -2.431658

H -1.847091 3.136506 -0.712506

C -0.787766 1.055143 -2.295286

H -0.904834 0.034051 -2.665408

H -1.475692 1.718560 -2.828885

C 1.356025 0.568700 -1.328887

H 2.223336 1.031231 -0.838943

C 0.244513 0.357803 -0.269415

H 0.584374 0.651025 0.732088

H 1.166663 1.581473 -3.295851

H -1.901711 0.898184 -0.341154

C 1.669735 3.535590 -0.949247

C 0.086080 3.027990 0.687393

O 2.726895 3.910590 -1.409130

O -0.287761 2.868601 1.843635

N 1.337350 3.603002 0.394015

C 2.248141 4.101954 1.397862

H 3.038324 3.372309 1.604404

H 2.711168 5.022880 1.037767

H 1.674855 4.286356 2.307012

O -3.071421 3.264232 -0.772900

C -3.433253 4.160323 0.244547

H -4.310513 3.791090 0.796816

H -2.615486 4.291321 0.971792

H -3.688287 5.149757 -0.161226

C -4.224272 0.470209 -1.659589

H -4.119344 1.202376 -2.470144

H -3.382525 -0.243313 -1.738976

H -5.157343 -0.084965 -1.810863

O -4.243465 1.112140 -0.411975

H -3.737803 2.038966 -0.532338

C -2.837421 0.725603 2.426776

H -2.337601 0.229958 3.267509

H -2.143441 1.454422 1.988214

H -3.715676 1.253572 2.816574

C -2.638286 -3.582580 2.652355

H -1.895797 -4.369978 2.800400

H -3.109558 -3.358879 3.616164

H -3.408691 -3.947428 1.962765

O -1.968867 -2.447342 2.135688

H -2.621416 -1.730688 1.890069

O -3.216503 -0.279584 1.499875

H -3.669211 0.189227 0.725430

C 0.679382 0.215280 3.240815

H 1.585564 0.545084 2.725890

H -0.059257 1.017351 3.172223

H 0.928849 -0.003293 4.283932

O 0.243419 -0.974132 2.583922

H -0.547641 -1.356042 3.005763

10(WB97X-D)

SCF done: -1742.049748

thermal Free Energies -1741.648414

Pd 2.693249 0.129844 -0.129044

P 4.846983 -0.873382 0.589787

C 4.743068 -1.383283 2.360931

H 3.772522 -1.867824 2.499584

H 5.559267 -2.057303 2.642715

H 4.773004 -0.493351 2.998869

C 5.185744 -2.465683 -0.276217

H 5.493688 -2.255183 -1.305862

H 5.968835 -3.045047 0.225622

H 4.235537 -3.009358 -0.313864

C 6.497466 -0.024693 0.524767

H 6.456615 0.917604 1.081663

H 7.289896 -0.652383 0.947125

H 6.752510 0.206247 -0.515126

C 3.556159 4.127315 2.158874

C 3.694575 3.030031 1.315753

C 3.395706 3.132511 -0.046511

C 2.966853 4.366056 -0.545303

C 2.824437 5.464829 0.297738

C 3.117790 5.348731 1.652770

H 3.787072 4.026266 3.216692

H 4.016172 2.070641 1.716334

H 2.749845 4.466181 -1.606505

H 2.485480 6.414193 -0.109415

H 3.008417 6.206219 2.312161

C 3.604720 1.983773 -0.983027

H 4.649351 1.743532 -1.182189

C 2.670280 1.493918 -1.872472

H 3.024166 0.872417 -2.695576

C -1.120532 1.515613 -2.755994

C 0.376472 1.205000 -2.966722

C -0.187856 -0.119763 -1.206108

C -1.439542 0.719582 -1.489607

H -1.673825 1.199879 -3.654349

H -3.144089 -0.514815 -1.425612

C 0.364123 -0.304682 -2.630975

H 1.347122 -0.788319 -2.637811

H -0.323199 -0.858775 -3.284773

C 1.182001 1.782272 -1.789057

H 0.975650 2.846110 -1.629125

C 0.836449 0.856171 -0.605009

H 0.489874 1.375053 0.294565

H 0.765253 1.486535 -3.951558

H -0.338549 -1.042052 -0.646193

C -1.579277 2.932618 -2.452434

C -1.950941 1.621268 -0.549411

O -1.514777 3.931045 -3.149746

O -2.207942 1.521045 0.679216

N -2.179497 2.880946 -1.209436

C -2.692503 4.048484 -0.546100

H -3.775596 4.175116 -0.705185

H -2.491728 3.957687 0.525087

H -2.197416 4.933113 -0.954630

O -3.977411 -0.785119 -0.962944

C -3.575511 -1.612126 0.085887

O -2.590349 -2.352302 -0.136575

O -4.227356 -1.482005 1.145076

Cs -1.078725 -1.065060 2.211611

C 1.575566 -2.396544 0.592705

O 2.072603 -2.604123 -0.566024

O 0.823669 -3.213544 1.205126

O 1.802453 -1.218559 1.184442

Cs -0.473314 -3.975505 -1.359367

Cs -5.188182 1.301457 0.763706

TS11-12(WB97X-D)

Pd 0.232706 -1.770872 0.530100

P 0.294236 -3.223532 -1.275806

C -0.623681 -4.706214 -0.649936

H -1.676988 -4.452300 -0.500666

H -0.548727 -5.538616 -1.358250

H -0.209226 -5.021516 0.312678

C -0.452911 -3.033927 -2.954393

H 0.111166 -2.316771 -3.556038

H -0.444124 -4.003842 -3.463130

H -1.485327 -2.683245 -2.867245

C 1.950272 -3.944521 -1.657653

H 2.403559 -4.311594 -0.732159

H 1.859928 -4.772367 -2.369056

H 2.604095 -3.172948 -2.069808

C 6.254765 0.972622 0.421051

C 5.518911 0.442731 -0.632157

C 4.211287 -0.015292 -0.433767

C 3.673890 0.038792 0.856883

C 4.411335 0.564199 1.913725

C 5.701001 1.039371 1.697695

H 7.264038 1.336174 0.245784

H 5.954792 0.394950 -1.628068

H 2.679149 -0.366212 1.035129

H 3.979639 0.595275 2.911739

H 6.276758 1.451788 2.522103

C 3.444671 -0.565610 -1.574154

H 4.008929 -1.214889 -2.245745

C 2.163485 -0.320339 -1.870416

H 1.747383 -0.811340 -2.752870

C -0.262935 2.655287 -1.646859

C 0.340269 1.374815 -2.246460

C -1.128131 0.566933 -0.709893

C -1.176212 2.109425 -0.542051

H -0.811596 3.196600 -2.429260

H -2.475382 2.315268 -0.889091

C -0.913940 0.478736 -2.230814

H -0.728568 -0.538676 -2.586000

H -1.752718 0.913661 -2.783599

C 1.255771 0.678466 -1.199814

H 1.901456 1.407364 -0.692718

C 0.227024 0.153031 -0.167469

H 0.430449 0.520920 0.844807

H 0.833092 1.515472 -3.213065

H -1.995871 0.050342 -0.288115

C 0.654688 3.645116 -0.955962

C -0.700522 2.715104 0.702841

O 1.542442 4.323455 -1.432244

O -1.002562 2.481038 1.869290

N 0.278760 3.677696 0.375775

C 0.933887 4.518405 1.349485

H 2.000432 4.278905 1.409258

H 0.830958 5.571354 1.071831

H 0.456280 4.333959 2.313377

O -3.583938 2.321003 -1.409194

C -4.172231 3.571736 -1.221890

H -4.573860 3.976773 -2.164647

H -5.001149 3.546257 -0.493742

H -3.434601 4.301611 -0.841057

C -4.362845 -0.733177 -2.223171

H -4.394268 -0.189419 -3.176883

H -3.327926 -1.089624 -2.071116

H -5.016188 -1.609181 -2.303623

O -4.815105 0.070259 -1.163727

H -4.402740 0.989980 -1.270032

C -3.908595 0.951947 1.788859

H -2.971538 1.099738 2.333648

H -3.914052 1.659856 0.952472

H -4.759336 1.162264 2.451115

C -3.199430 -2.122461 3.861660

H -4.157711 -2.608210 3.644438

H -2.639541 -2.746567 4.563548

H -3.398578 -1.149460 4.330801

O -2.423477 -1.987522 2.683145

H -2.956188 -1.434808 2.049424

O -3.969771 -0.386429 1.307510

H -4.349056 -0.362224 0.391733

C 0.445900 -0.051639 3.340061

H 1.510960 0.113559 3.152052

H -0.125177 0.818412 2.995090

H 0.300668 -0.193339 4.416570

O 0.066839 -1.241536 2.648662

H -0.902423 -1.437493 2.800524

**12**

SCF done: -1733.022052

thermal Free Energies -1732.362389

Pd 0.854877 -0.728099 0.339874

P 2.235965 -0.286265 2.118359

C 3.922209 -0.915797 1.668910

H 4.290552 -0.339412 0.816238

H 4.609120 -0.805139 2.515360

H 3.861074 -1.963242 1.369576

C 2.645367 1.400730 2.768968

H 1.775469 1.866930 3.234819

H 3.440148 1.304813 3.517526

H 2.988783 2.022635 1.935167

C 1.816898 -1.249584 3.654141

H 1.671833 -2.301176 3.392014

H 2.619942 -1.168105 4.395240

H 0.889076 -0.871025 4.093348

C -5.664123 -3.254106 -0.246834

C -4.751061 -2.936518 0.756532

C -3.535709 -2.293561 0.457346

C -3.244446 -2.016221 -0.890612

C -4.158295 -2.340361 -1.894907

C -5.373082 -2.951489 -1.579312

H -6.601607 -3.740482 0.010383

H -4.982850 -3.176029 1.792035

H -2.280155 -1.595163 -1.157297

H -3.911027 -2.126647 -2.931934

H -6.081080 -3.202728 -2.364646

C -2.608217 -1.980258 1.564544

H -2.554028 -2.750870 2.334981

C -1.873116 -0.871558 1.773502

H -1.268141 -0.857907 2.679932

C -2.556068 2.882643 1.047460

C -2.221246 1.627369 1.902509

C -0.197857 2.208802 1.037452

C -1.184125 3.265684 0.429539

H -2.977494 3.663140 1.689781

H -0.856504 4.278634 0.689446

C -0.832456 2.039184 2.437693

H -0.360405 1.251387 3.032447

H -0.852668 2.961811 3.030413

C -1.867494 0.413082 0.980326

H -2.604957 0.313487 0.178728

C -0.471052 0.843516 0.389508

H -0.520277 0.933963 -0.701434

H -2.988773 1.395859 2.644672

H 0.841145 2.531845 0.980373

C -3.487489 2.682790 -0.137283

C -1.402764 3.208205 -1.076807

O -4.670037 2.408078 -0.129725

O -0.590287 3.406668 -1.962194

N -2.741373 2.881146 -1.304033

C -3.318994 2.734761 -2.630319

H -4.228127 3.336906 -2.703935

H -2.576580 3.071859 -3.354786

H -3.579127 1.688469 -2.819579

O 3.319907 2.691516 -0.124143

C 2.926138 3.752738 -0.982331

H 1.929995 3.597316 -1.418122

H 2.897998 4.667253 -0.379751

H 3.647541 3.911236 -1.798836

C 5.500777 0.902948 -2.068107

H 6.242239 0.339510 -1.484309

H 5.685203 0.718101 -3.136965

H 5.647438 1.969082 -1.871299

O 4.170757 0.575709 -1.695979

H 3.471483 1.890075 -0.671354

C 4.061349 -2.861827 -2.774312

H 4.076351 -3.942615 -2.572693

H 3.198286 -2.645289 -3.423361

H 4.973038 -2.607254 -3.324532

C 1.678563 -3.776109 0.425252

H 0.629218 -3.965661 0.703345

H 2.026428 -4.642539 -0.160658

H 2.268858 -3.763070 1.359535

O 1.825820 -2.586713 -0.309858

H 3.207712 -2.346269 -1.053250

O 4.043234 -2.109304 -1.574640

H 4.084806 -0.414384 -1.674186

C 0.592637 -0.692159 -2.828944

H 0.114777 0.290343 -2.857859

H 1.676958 -0.553856 -2.751473

H 0.351657 -1.231314 -3.753443

O 0.070714 -1.404896 -1.697639

H 0.693547 -2.161870 -1.461107

**TS12-13**

SCF done: -1732.763383

thermal Free Energies -1732.399313

Pd 1.647300 -0.874800 -0.170800

P 3.518100 -2.232000 0.105200

C 3.104500 -4.020500 -0.143400

H 2.205300 -4.211500 0.447300

H 2.879100 -4.204500 -1.198100

H 3.922700 -4.676000 0.174400

C 5.039500 -1.968400 -0.929900

H 5.385400 -0.937400 -0.808800

H 5.843000 -2.656100 -0.644300

H 4.794200 -2.127400 -1.984800

C 4.185400 -2.193100 1.837300

H 3.369600 -2.426200 2.526500

H 4.994100 -2.920900 1.968200

H 4.561100 -1.191100 2.064900

C 5.391700 2.828100 -1.089800

C 4.486800 1.884400 -1.574800

C 3.257700 1.642900 -0.931100

C 2.978000 2.387600 0.231200

C 3.878800 3.336300 0.713900

C 5.090800 3.563100 0.058100

H 6.329600 2.992900 -1.613900

H 4.726400 1.329400 -2.479400

H 2.058800 2.212100 0.779900

H 3.633700 3.896300 1.612700

H 5.791400 4.301100 0.438600

C 2.351100 0.610100 -1.503100

H 2.812200 0.086600 -2.340900

C 0.918200 0.764000 -1.707200

H 0.523300 0.112300 -2.477600

C -2.364500 2.414400 -0.650300

C -1.434000 1.858000 -1.772600

C -1.522100 0.143900 -0.265400

C -2.418300 1.252500 0.383600

H -3.354600 2.642200 -1.056900

H -3.439200 0.886400 0.530200

C -1.892700 0.378000 -1.748800

H -1.371900 -0.284500 -2.444300

H -2.964300 0.262000 -1.929200

C -0.007000 1.795500 -1.189500

H 0.438100 2.740000 -0.880800

C -0.082900 0.651700 -0.180800

H 0.346100 0.861400 0.799800

H -1.517000 2.390500 -2.722700

H -1.694100 -0.856300 0.124400

C -1.861800 3.617200 0.132900

C -1.927000 1.857700 1.684900

O -1.641500 4.743900 -0.267500

O -1.764400 1.303300 2.756900

N -1.668400 3.215000 1.457900

C -1.196200 4.124600 2.488200

H -1.910600 4.941400 2.626400

H -1.096000 3.552300 3.411400

H -0.232200 4.554700 2.200800

O -5.553700 0.894800 -0.177900

C -6.931300 1.158000 -0.011500

H -7.486900 0.288800 0.376500

H -7.030400 1.970500 0.715900

H -7.416100 1.481800 -0.947000

C -5.801600 -2.158100 -2.411000

H -6.514700 -2.635300 -1.722700

H -6.366400 -1.582100 -3.150600

H -5.249600 -2.948800 -2.938700

O -4.924200 -1.265800 -1.744000

H -5.446200 0.141400 -0.804200

C -4.019800 -2.156400 1.554500

H -4.275600 -2.990700 2.224100

H -3.182100 -1.597700 1.993100

H -4.877500 -1.480200 1.490100

C -1.155700 -4.974400 0.592300

H -0.243300 -5.464200 0.220500

H -1.142200 -5.017400 1.692900

H -2.017100 -5.551600 0.239100

O -1.276900 -3.653400 0.102000

H -2.820100 -3.007900 0.202100

O -3.734700 -2.612800 0.238900

H -4.422200 -1.777700 -1.054700

C 0.302900 -1.867000 2.376000

H 1.173900 -1.632700 3.014900

H -0.343900 -0.973500 2.374600

H -0.260700 -2.666800 2.887200

O 0.682600 -2.297400 1.092900

H -0.501700 -3.094900 0.462600

**13**

SCF done: -1732.775833

thermal Free Energies -1732.402524

Pd 1.615907 -1.158573 0.150730

P 3.549751 -2.437534 0.357537

C 3.200653 -4.242044 0.126544

H 2.332858 -4.468151 0.750827

H 2.943569 -4.437821 -0.918792

H 4.056336 -4.862696 0.414029

C 5.019123 -2.114544 -0.734285

H 5.326838 -1.069944 -0.627326

H 5.860284 -2.768167 -0.478416

H 4.741219 -2.286221 -1.779071

C 4.279428 -2.366889 2.063287

H 3.500280 -2.631436 2.782884

H 5.121548 -3.060705 2.164428

H 4.622072 -1.349791 2.275641

C 5.248433 2.568441 -0.795333

C 4.343572 1.624698 -1.280385

C 3.114445 1.383228 -0.636660

C 2.834690 2.127864 0.525620

C 3.735543 3.076640 1.008368

C 4.947507 3.303445 0.352601

H 6.186368 2.733171 -1.319468

H 4.583108 1.069713 -2.184947

H 1.915549 1.952445 1.074385

H 3.490445 3.636559 1.907128

H 5.648131 4.041431 0.733060

C 2.207844 0.350415 -1.208600

H 2.668920 -0.173122 -2.046454

C 0.774960 0.504329 -1.412749

H 0.380022 -0.147369 -2.183120

C -2.441233 2.393909 -0.639559

C -1.510668 1.837490 -1.761862

C -1.598787 0.123417 -0.254732

C -2.494958 1.231946 0.394306

H -3.431278 2.621679 -1.046242

H -3.515926 0.865850 0.540878

C -1.969388 0.357434 -1.738057

H -1.448598 -0.305019 -2.433653

H -3.040965 0.241522 -1.918459

C -0.083652 1.775021 -1.178850

H 0.361463 2.719517 -0.870075

C -0.159590 0.631163 -0.170120

H 0.269414 0.840844 0.810458

H -1.593724 2.370019 -2.712030

H -1.770745 -0.876865 0.135079

C -1.938464 3.596648 0.143624

C -2.003712 1.837172 1.695591

O -1.718182 4.723418 -0.256791

O -1.841096 1.282733 2.767542

N -1.745077 3.194490 1.468565

C -1.272915 4.104084 2.498907

H -1.987297 4.920857 2.637120

H -1.172649 3.531754 3.422132

H -0.308857 4.534190 2.211457

O -5.630401 0.874293 -0.167224

C -7.008007 1.137487 -0.000760

H -7.563577 0.268330 0.387233

H -7.107120 1.949933 0.726573

H -7.492795 1.461318 -0.936275

C -5.878332 -2.178600 -2.400355

H -6.591380 -2.655852 -1.712026

H -6.443074 -1.602648 -3.139929

H -5.326282 -2.969362 -2.928004

O -5.000912 -1.286315 -1.733321

H -5.522915 0.120920 -0.793535

C -4.096485 -2.176915 1.565214

H -4.352302 -3.011180 2.234758

H -3.258755 -1.618189 2.003775

H -4.954153 -1.500671 1.500836

C -1.232404 -4.994904 0.602969

H -0.319949 -5.484742 0.231160

H -1.218881 -5.037955 1.703553

H -2.093738 -5.572139 0.249787

O -1.353544 -3.673883 0.112672

H -2.896797 -3.028421 0.212742

O -3.811404 -2.633271 0.249609

H -4.498897 -1.798246 -1.043957

C 0.226250 -1.887472 2.386709

H 1.097247 -1.653176 3.025587

H -0.420583 -0.993994 2.385296

H -0.337361 -2.687309 2.897909

O 0.605910 -2.317894 1.103578

H -0.578370 -3.115375 0.473311

**10’**

SCF done: -1742.414574

thermal Free Energies -1742.023562

Pd -3.313898 -0.504460 -0.039723

P -5.452776 0.152153 -0.495235

C -5.704419 0.832897 -2.206478

H -5.336847 0.101539 -2.932362

H -6.762334 1.040320 -2.404620

H -5.123144 1.752743 -2.316046

C -6.578099 -1.324552 -0.461875

H -6.671820 -1.678624 0.569204

H -7.572283 -1.089303 -0.858171

H -6.112654 -2.121343 -1.048210

C -6.345548 1.392069 0.563113

H -5.823308 2.351414 0.512964

H -7.378582 1.526859 0.223995

H -6.349484 1.049058 1.602126

C -2.209371 3.371172 -2.424869

C -2.184184 2.327461 -1.500028

C -2.754730 2.463084 -0.217782

C -3.357422 3.699542 0.084189

C -3.395017 4.745726 -0.841965

C -2.817906 4.588657 -2.102500

H -1.763706 3.230328 -3.407269

H -1.743918 1.372389 -1.773806

H -3.793917 3.844082 1.070527

H -3.870582 5.686012 -0.573062

H -2.844721 5.399156 -2.826156

C -2.747076 1.347223 0.792603

H -3.490143 1.580321 1.566982

C -1.430176 1.084282 1.515681

H -1.533479 0.232180 2.180065

C 1.844975 2.618276 2.632623

C 0.486048 2.001637 3.110401

C 1.151447 0.590724 1.433759

C 2.306601 1.651319 1.495101

H 2.551701 2.675560 3.468266

H 3.232728 1.090485 1.673385

C 0.856386 0.509575 2.953332

H 0.048054 -0.174411 3.227425

H 1.747725 0.230987 3.525495

C -0.540651 2.230470 1.991288

H -0.857701 3.248683 1.780836

C -0.087584 1.293192 0.873242

H -0.051869 1.642879 -0.154723

H 0.186858 2.333676 4.108369

H 1.466747 -0.347491 0.970118

C 1.748963 3.987640 1.986226

C 2.489991 2.553386 0.291101

O 1.421046 5.042535 2.492868

O 2.916837 2.272245 -0.823599

N 2.126286 3.849026 0.642084

C 2.131306 4.956086 -0.297965

H 1.855022 5.854495 0.255761

H 3.125332 5.074899 -0.739625

H 1.410101 4.775887 -1.100908

O 4.166310 -0.937593 0.882990

C 3.449872 -1.627571 0.056096

O 2.421591 -2.311252 0.481163

O 3.705841 -1.614652 -1.227393

Cs 0.865738 -2.094254 -2.027855

C -2.124748 -2.722096 -0.409998

O -3.370548 -2.537010 -0.751147

O -1.473716 -3.777277 -0.573136

O -1.546164 -1.645696 0.140958

Cs 0.290776 -3.357359 2.026405

Cs 5.566061 0.577891 -1.138783

**TS10-11’**

SCF done: -1742.393755

Pd 3.347656 -0.460216 0.081442

P 5.520506 0.121858 0.429116

C 5.815512 0.894299 2.093658

H 5.435624 0.217023 2.864288

H 6.881261 1.082220 2.267997

H 5.261139 1.835110 2.154416

C 6.589841 -1.395462 0.480994

H 6.652734 -1.824695 -0.523612

H 7.598966 -1.171129 0.844612

H 6.105328 -2.128036 1.132424

C 6.444052 1.259760 -0.713358

H 5.955301 2.238064 -0.719295

H 7.485014 1.378847 -0.393177

H 6.424028 0.853035 -1.728999

C 2.437189 3.673908 2.008450

C 2.394724 2.530945 1.208957

C 2.921611 2.526054 -0.097678

C 3.498590 3.725222 -0.558244

C 3.550383 4.870710 0.239936

C 3.017211 4.852039 1.529433

H 2.024724 3.641698 3.014640

H 1.974899 1.610469 1.605321

H 3.901610 3.759325 -1.568531

H 4.003605 5.779127 -0.149951

H 3.055542 5.740457 2.154456

C 2.899070 1.307446 -0.986193

H 3.673187 1.434239 -1.753736

C 1.594975 1.002776 -1.707492

H 1.757623 0.230589 -2.455271

C -1.943818 2.173076 -2.504102

C -0.537471 1.807556 -3.100056

C -0.899515 0.195456 -1.528139

C -2.157072 1.103551 -1.395488

H -2.684158 2.133556 -3.315863

H -3.205391 0.084795 -1.390149

C -0.700954 0.268097 -3.061196

H 0.164038 -0.281703 -3.445928

H -1.592079 -0.069662 -3.600292

C 0.531739 2.071341 -2.026324

H 0.735345 3.100232 -1.740852

C 0.284251 0.995524 -0.983597

H 0.281070 1.246256 0.072607

H -0.348196 2.254177 -4.080686

H -1.045386 -0.801628 -1.123579

C -2.114104 3.514531 -1.811379

C -2.321329 1.856478 -0.168826

O -2.073705 4.638166 -2.286433

O -2.439488 1.466890 1.014153

N -2.401136 3.238346 -0.472848

C -2.586731 4.274074 0.521009

H -2.292168 5.222937 0.068251

H -3.636617 4.349281 0.838947

H -1.974547 4.061105 1.401305

O -4.026671 -0.748885 -1.135764

C -3.560302 -1.697119 -0.273915

O -2.541488 -2.385598 -0.608416

O -4.145169 -1.800332 0.852274

Cs -1.141395 -1.428042 1.975156

C 2.023758 -2.501774 0.870825

O 3.292320 -2.356242 1.129183

O 1.308443 -3.465306 1.236461

O 1.494382 -1.510779 0.145530

Cs -0.086203 -3.643522 -1.556400

Cs -5.547364 0.783268 0.884843

**11’**

SCF done: -1742.400027

thermal Free Energies -1742.009787

Pd -3.361488 -0.428589 -0.074877

P -5.529519 0.186416 -0.409022

C -5.820933 0.982446 -2.063248

H -5.445751 0.313851 -2.843644

H -6.885780 1.178765 -2.234067

H -5.262030 1.921188 -2.111946

C -6.617135 -1.317333 -0.475835

H -6.682652 -1.757620 0.523821

H -7.624421 -1.076319 -0.833812

H -6.143819 -2.048201 -1.137313

C -6.439881 1.322013 0.746730

H -5.947723 2.298520 0.753390

H -7.482429 1.447505 0.434201

H -6.414522 0.910771 1.760365

C -2.233244 3.561711 -2.106821

C -2.231391 2.460638 -1.250601

C -2.837872 2.514052 0.020235

C -3.449590 3.728822 0.384645

C -3.461660 4.832751 -0.471702

C -2.851106 4.755894 -1.724009

H -1.758744 3.484979 -3.082841

H -1.780947 1.526610 -1.574242

H -3.910209 3.810392 1.367188

H -3.943059 5.755009 -0.155006

H -2.856770 5.611989 -2.393625

C -2.867313 1.338592 0.963794

H -3.655687 1.519628 1.705104

C -1.594134 1.032121 1.738923

H -1.801976 0.309523 2.524879

C 1.907829 2.256778 2.631731

C 0.476951 1.922084 3.178736

C 0.928626 0.209783 1.733136

C 2.143503 1.147988 1.589028

H 2.602752 2.238588 3.486766

H 3.532639 -0.593559 1.557347

C 0.642033 0.380139 3.246741

H -0.243741 -0.145722 3.617935

H 1.499982 0.084505 3.860409

C -0.533828 2.108004 2.039400

H -0.710844 3.115080 1.670913

C -0.244650 0.968477 1.083087

H -0.178618 1.165020 0.018427

H 0.232431 2.423669 4.120035

H 1.080851 -0.813101 1.387778

C 2.192343 3.559177 1.891500

C 2.448272 1.779083 0.372396

O 2.158737 4.709320 2.310408

O 2.574111 1.343667 -0.814027

N 2.615039 3.191530 0.622097

C 2.978540 4.155226 -0.388792

H 4.071448 4.281426 -0.469880

H 2.591379 3.827794 -1.357243

H 2.552925 5.122095 -0.109982

O 4.228802 -1.095776 1.064284

C 3.597684 -1.955772 0.149316

O 2.516425 -2.487694 0.515872

O 4.179735 -2.060479 -0.954953

Cs 1.037431 -1.229602 -1.921003

C -2.064401 -2.475495 -0.894422

O -1.523365 -1.496632 -0.157318

O -1.351314 -3.431663 -1.283392

O -3.334593 -2.326146 -1.133595

Cs -0.014724 -3.665473 1.505376

Cs 5.519677 0.673728 -0.966228

**12’**

SCF done: -1733.0220524

thermal Free Energies -1732.397031

Pd 0.854877 -0.728099 0.339874

P 2.235965 -0.286265 2.118359

C 3.922209 -0.915797 1.668910

H 4.290552 -0.339412 0.816238

H 4.609120 -0.805139 2.515360

H 3.861074 -1.963242 1.369576

C 2.645367 1.400730 2.768968

H 1.775469 1.866930 3.234819

H 3.440148 1.304813 3.517526

H 2.988783 2.022635 1.935167

C 1.816898 -1.249584 3.654141

H 1.671833 -2.301176 3.392014

H 2.619942 -1.168105 4.395240

H 0.889076 -0.871025 4.093348

C -5.664123 -3.254106 -0.246834

C -4.751061 -2.936518 0.756532

C -3.535709 -2.293561 0.457346

C -3.244446 -2.016221 -0.890612

C -4.158295 -2.340361 -1.894907

C -5.373082 -2.951489 -1.579312

H -6.601607 -3.740482 0.010383

H -4.982850 -3.176029 1.792035

H -2.280155 -1.595163 -1.157297

H -3.911027 -2.126647 -2.931934

H -6.081080 -3.202728 -2.364646

C -2.608217 -1.980258 1.564544

H -2.554028 -2.750870 2.334981

C -1.873116 -0.871558 1.773502

H -1.268141 -0.857907 2.679932

C -2.556068 2.882643 1.047460

C -2.221246 1.627369 1.902509

C -0.197857 2.208802 1.037452

C -1.184125 3.265684 0.429539

H -2.977494 3.663140 1.689781

H -0.856504 4.278634 0.689446

C -0.832456 2.039184 2.437693

H -0.360405 1.251387 3.032447

H -0.852668 2.961811 3.030413

C -1.867494 0.413082 0.980326

H -2.604957 0.313487 0.178728

C -0.471052 0.843516 0.389508

H -0.520277 0.933963 -0.701434

H -2.988773 1.395859 2.644672

H 0.841145 2.531845 0.980373

C -3.487489 2.682790 -0.137283

C -1.402764 3.208205 -1.076807

O -4.670037 2.408078 -0.129725

O -0.590287 3.406668 -1.962194

N -2.741373 2.881146 -1.304033

C -3.318994 2.734761 -2.630319

H -4.228127 3.336906 -2.703935

H -2.576580 3.071859 -3.354786

H -3.579127 1.688469 -2.819579

O 3.319907 2.691516 -0.124143

C 2.926138 3.752738 -0.982331

H 1.929995 3.597316 -1.418122

H 2.897998 4.667253 -0.379751

H 3.647541 3.911236 -1.798836

C 5.500777 0.902948 -2.068107

H 6.242239 0.339510 -1.484309

H 5.685203 0.718101 -3.136965

H 5.647438 1.969082 -1.871299

O 4.170757 0.575709 -1.695979

H 3.471483 1.890075 -0.671354

C 4.061349 -2.861827 -2.774312

H 4.076351 -3.942615 -2.572693

H 3.198286 -2.645289 -3.423361

H 4.973038 -2.607254 -3.324532

C 1.678563 -3.776109 0.425252

H 0.629218 -3.965661 0.703345

H 2.026428 -4.642539 -0.160658

H 2.268858 -3.763070 1.359535

O 1.825820 -2.586713 -0.309858

H 3.207712 -2.346269 -1.053250

O 4.043234 -2.109304 -1.574640

H 4.084806 -0.414384 -1.674186

C 0.592637 -0.692159 -2.828944

H 0.114777 0.290343 -2.857859

H 1.676958 -0.553856 -2.751473

H 0.351657 -1.231314 -3.753443

O 0.070714 -1.404896 -1.697639

H 0.693547 -2.161870 -1.461107

**TS12-13’**

SCF done: -1733.007245

thermal Free Energies -1742.009787

Pd -3.361488 -0.428589 -0.074877

P -5.529519 0.186416 -0.409022

C -5.820933 0.982446 -2.063248

H -5.445751 0.313851 -2.843644

H -6.885780 1.178765 -2.234067

**13’**

SCF done: -1732.969058

thermal Free Energies -1732.362389

Pd 0.168142 -1.830772 0.332739

P 0.569155 -3.390640 -1.301276

C -0.477997 -4.890126 -0.923341

H -1.535356 -4.623191 -1.006960

H -0.255463 -5.715633 -1.610077

H -0.289014 -5.215342 0.103868

C 0.224971 -3.187614 -3.122545

H 0.920486 -2.468939 -3.563594

H 0.336210 -4.147508 -3.639619

H -0.794015 -2.817424 -3.265030

C 2.278747 -4.130418 -1.322133

H 2.512425 -4.510390 -0.323210

H 2.351766 -4.948673 -2.047899

H 3.007956 -3.354277 -1.566297

C 6.481450 0.912339 1.178494

C 5.832218 0.341980 0.085864

C 4.451390 0.072392 0.119266

C 3.750901 0.358325 1.305092

C 4.401300 0.925712 2.400981

C 5.766474 1.211532 2.340987

H 7.546476 1.122186 1.124200

H 6.394521 0.112517 -0.816453

H 2.699302 0.099438 1.378368

H 3.841157 1.135023 3.308810

H 6.271521 1.652997 3.195831

C 3.811613 -0.531237 -1.064565

H 4.450887 -1.221992 -1.616516

C 2.580488 -0.318079 -1.563559

H 2.310992 -0.882882 -2.455535

C 0.242476 2.717684 -1.874689

C 0.986179 1.460084 -2.376398

C -0.881135 0.591089 -1.345553

C -0.855316 2.092604 -1.031480

H -0.126936 3.275653 -2.749190

H -2.666353 2.477007 -1.535282

C -0.234248 0.576290 -2.748443

H 0.038631 -0.428411 -3.081291

H -0.870981 1.038435 -3.510622

C 1.570252 0.716155 -1.133032

H 2.079579 1.422902 -0.468429

C 0.277246 0.202613 -0.445840

H 0.174296 0.485873 0.603030

H 1.718898 1.638189 -3.167789

H -1.821036 0.055645 -1.215956

C 0.906914 3.740314 -0.955491

C -0.818496 2.704392 0.272788

O 1.839025 4.490900 -1.192602

O -1.455800 2.473660 1.311060

N 0.174792 3.735053 0.222964

C 0.451608 4.630849 1.327632

H 0.336641 5.674072 1.014684

H -0.257356 4.394985 2.123102

H 1.476402 4.493875 1.688454

O -3.651342 2.542643 -1.697304

C -4.148563 3.623217 -0.911658

H -5.241171 3.592331 -0.962165

H -3.833932 3.545190 0.136237

H -3.813362 4.590292 -1.311538

C -5.470152 -0.340211 -1.997945

H -6.389325 0.248103 -1.859133

H -5.144642 -0.234768 -3.041794

H -5.703567 -1.392548 -1.811425

O -4.443900 0.042179 -1.094716

H -4.186097 0.984065 -1.288239

C -4.489533 0.940851 2.156429

H -4.469393 0.812370 3.243131

H -3.538483 1.380427 1.831415

H -5.310253 1.629949 1.911550

C -3.502146 -2.702254 3.628492

H -2.771741 -3.409774 4.031888

H -3.741686 -1.964926 4.409070

H -4.418711 -3.252956 3.378506

O -2.933600 -2.100498 2.475088

H -3.593433 -1.452862 2.093222

O -4.718323 -0.345614 1.577860

H -4.653578 -0.245723 0.593274

C -0.209999 -0.092484 3.194266

H 0.870207 0.049818 3.289024

H -0.655340 0.785891 2.709917

H -0.632198 -0.207063 4.200393

O -0.422584 -1.288294 2.438378

H -1.387950 -1.548043 2.494652

**14**

SCF done: -1270.070559

thermal Free Energies -1269.651274

Pd 2.333028 -1.099534 0.123942

P 2.808017 0.705175 1.529970

C 1.490997 1.410581 2.641219

H 1.014287 0.602775 3.203734

H 1.917724 2.136524 3.342262

H 0.730498 1.908803 2.035149

C 4.094004 0.180055 2.777103

H 4.993934 -0.158162 2.254390

H 4.358802 1.004422 3.448977

H 3.707594 -0.654339 3.370356

C 3.580980 2.236597 0.811829

H 2.877777 2.716220 0.127202

H 3.858020 2.942465 1.603010

H 4.476316 1.957566 0.248693

C 1.830714 2.915297 -2.696534

C 1.738906 1.558335 -2.402535

C 0.859444 1.067140 -1.412676

C 0.064646 2.015558 -0.737179

C 0.151475 3.377833 -1.033308

C 1.035328 3.838496 -2.010344

H 2.518085 3.253755 -3.467499

H 2.360937 0.849592 -2.943786

H -0.642160 1.695917 0.018507

H -0.482666 4.081027 -0.499172

H 1.098108 4.898466 -2.240235

C 0.837979 -0.405253 -1.186989

H 1.256616 -0.891575 -2.070998

C -0.385189 -1.191211 -0.777825

H -0.164315 -2.243376 -0.912101

C -4.142567 -1.164718 -0.365333

C -2.940159 -1.729430 -1.193602

C -2.221319 -1.912730 0.963871

C -3.643712 -1.273491 1.108067

H -5.044334 -1.753785 -0.563276

H -4.283096 -1.904559 1.734723

C -2.503781 -2.858468 -0.228021

H -1.641641 -3.437145 -0.562103

H -3.318372 -3.559243 -0.015214

C -1.800553 -0.707209 -1.082628

H -1.923750 0.257510 -1.565411

C -1.318284 -0.831084 0.356668

H -1.100934 0.030225 0.979703

H -3.210538 -2.028277 -2.208835

H -1.852206 -2.378000 1.880536

C -4.476921 0.305268 -0.565202

C -3.699042 0.150525 1.643747

O -4.901343 0.845381 -1.567221

O -3.365684 0.555201 2.741280

N -4.212521 0.971173 0.637910

C -4.402987 2.401108 0.813426

H -4.928040 2.774111 -0.066993

H -4.987361 2.588515 1.717823

H -3.438330 2.909546 0.911509

O 2.173574 -2.888103 -0.753009

C 2.802436 -3.165686 -1.965932

H 2.379444 -2.591215 -2.816730

H 3.892513 -2.979110 -1.960148

H 2.658456 -4.230835 -2.215641

**TS14-16**

SCF done: -1168.201485

thermal Free Energies -1269.627174

Pd -1.749200 -0.338100 0.233900

P -3.373700 -1.888600 -0.492100

C -3.064700 -3.127900 -1.844900

H -2.228300 -3.775600 -1.564100

H -3.949100 -3.748300 -2.028400

H -2.798700 -2.602100 -2.767000

C -3.958700 -2.906900 0.938400

H -4.136800 -2.197200 1.750600

H -4.872600 -3.460300 0.696000

H -3.175600 -3.609400 1.239200

C -4.905700 -1.004800 -1.041700

H -4.707600 -0.452700 -1.965100

H -5.738500 -1.698600 -1.201800

H -5.143700 -0.296100 -0.244800

C -1.305300 4.804000 0.308600

C -1.019200 3.574500 0.900300

C -0.605000 2.474300 0.125800

C -0.493700 2.651000 -1.268900

C -0.782500 3.879100 -1.857700

C -1.188900 4.963200 -1.073300

H -1.620700 5.638600 0.929400

H -1.113300 3.457400 1.976500

H -0.188600 1.814900 -1.890500

H -0.690000 3.992200 -2.934900

H -1.411900 5.920700 -1.536000

C -0.294500 1.198300 0.793000

H -0.356300 1.204200 1.879900

C 0.449900 0.149000 0.204200

H -0.639000 -0.701600 -0.852400

C 3.558800 -1.972400 0.179600

C 2.021900 -1.991300 0.478400

C 2.150900 -0.932900 -1.536100

C 3.646600 -1.254000 -1.200800

H 3.949000 -2.995200 0.161000

H 4.079700 -1.878700 -1.988800

C 1.499600 -2.210800 -0.956000

H 0.413500 -2.240800 -1.030400

H 1.905600 -3.120700 -1.411800

C 1.620600 -0.547700 0.799400

H 2.027400 -0.117000 1.712600

C 1.717500 0.180400 -0.573300

H 2.196100 1.151100 -0.687300

H 1.726600 -2.727900 1.228600

H 1.972700 -0.724300 -2.593700

C 4.430500 -1.139700 1.109800

C 4.567700 -0.063100 -0.970600

O 4.641200 -1.312900 2.293700

O 4.904500 0.781100 -1.776600

N 4.980600 -0.093900 0.365300

C 5.858900 0.905200 0.953200

H 6.724600 0.418000 1.409700

H 6.176900 1.575100 0.153400

H 5.329100 1.469200 1.726800

O -3.293600 0.040300 1.554000

C -3.422600 1.258800 2.210600

H -2.615700 1.454000 2.946800

H -3.448900 2.134400 1.534300

H -4.370300 1.270300 2.780500

**16**

SCF done: -1168.225322

thermal Free Energies -1269.630226

Pd 2.154282 -1.490205 -0.519931

P 3.367961 -0.182779 1.249638

C 2.457871 0.560924 2.698253

H 1.879522 -0.216767 3.206069

H 3.151774 1.019462 3.411955

H 1.765500 1.322794 2.330453

C 4.634304 -1.240453 2.128185

H 5.337384 -1.651592 1.397125

H 5.191701 -0.662029 2.873699

H 4.132207 -2.075362 2.626795

C 4.415997 1.250877 0.684653

H 3.774420 2.010010 0.228985

H 4.971853 1.694164 1.518961

H 5.122889 0.900374 -0.073480

C 2.093609 3.019751 -2.126979

C 1.793192 1.670326 -2.294833

C 0.983940 0.967824 -1.375858

C 0.496024 1.693494 -0.268337

C 0.788638 3.048153 -0.102221

C 1.589754 3.721058 -1.027554

H 2.714589 3.528052 -2.860210

H 2.187082 1.137336 -3.156745

H -0.125027 1.200183 0.469718

H 0.382865 3.581655 0.753995

H 1.812705 4.776494 -0.897566

C 0.708291 -0.475385 -1.645541

H 1.003958 -0.688961 -2.673499

C -0.657221 -1.102070 -1.430624

H -0.716772 -2.021485 -2.002831

C -4.259140 -0.473100 -0.475672

C -3.288026 -0.876370 -1.636493

C -2.529434 -2.098901 0.134386

C -3.736517 -1.304070 0.735881

H -5.292649 -0.710275 -0.749934

H -4.483282 -1.995668 1.140637

C -3.105378 -2.370960 -1.276179

H -2.438307 -2.921911 -1.941211

H -4.059472 -2.907909 -1.238162

C -1.921854 -0.260029 -1.310454

H -1.829261 0.821130 -1.343148

C -1.415582 -1.076287 -0.125140

H -0.949435 -0.623938 0.744278

H -3.682405 -0.656042 -2.631218

H -2.241149 -2.972839 0.722791

C -4.207213 0.975874 -0.015974

C -3.407137 -0.271231 1.804492

O -4.519284 1.969424 -0.641804

O -2.944770 -0.456759 2.913766

N -3.729127 0.990095 1.299780

C -3.536779 2.210829 2.063948

H -3.949901 3.033627 1.478994

H -4.047248 2.131002 3.027367

H -2.471860 2.383893 2.249339

H 1.674194 -2.597800 -1.542670

**TS14-15**

SCF done: -1168.201485

thermal Free Energies -1269.631533

Pd 2.662200 0.231300 -0.672700

P 2.676400 -1.415800 1.067300

C 1.266700 -2.277900 1.919500

H 0.673900 -2.822400 1.181000

H 1.647800 -2.980800 2.668500

H 0.615300 -1.550100 2.410300

C 3.725800 -2.845200 0.503400

H 4.632000 -2.439100 0.046300

H 3.991200 -3.504000 1.338000

H 3.184700 -3.422200 -0.252700

C 3.664100 -0.743000 2.491900

H 3.093300 0.042400 2.996400

H 3.916900 -1.527500 3.214000

H 4.578700 -0.300500 2.088100

C -0.461900 4.169500 -0.040900

C -0.075700 3.068200 -0.805600

C 0.252100 1.834600 -0.211800

C 0.184200 1.761500 1.193700

C -0.208800 2.856500 1.962600

C -0.536200 4.068800 1.349600

H -0.709700 5.106200 -0.534100

H -0.027100 3.159500 -1.888500

H 0.461600 0.836400 1.689800

H -0.252500 2.764800 3.045200

H -0.837800 4.923900 1.948500

C 0.653900 0.674200 -1.082500

H 0.700100 1.035400 -2.113200

C -0.269500 -0.543600 -1.130500

H 0.190500 -1.371900 -1.662700

C -3.967700 -1.364200 -1.158900

C -2.652400 -1.344500 -2.009900

C -1.978100 -2.241200 -0.025500

C -3.503000 -1.969400 0.199700

H -4.738400 -1.958900 -1.660500

H -4.021900 -2.897300 0.462600

C -2.025200 -2.668000 -1.512300

H -1.051100 -2.897000 -1.949900

H -2.680700 -3.530200 -1.676900

C -1.743400 -0.276000 -1.395600

H -2.056600 0.762800 -1.461100

C -1.290200 -0.868300 -0.063400

H -1.318000 -0.271900 0.841900

H -2.829400 -1.259900 -3.084700

H -1.565500 -2.968900 0.677800

C -4.556000 -0.005800 -0.804300

C -3.841800 -0.909000 1.238000

O -5.041300 0.814700 -1.557600

O -3.633600 -0.943600 2.435800

N -4.449900 0.160200 0.580800

C -4.858700 1.373600 1.270100

H -5.518700 1.930000 0.603100

H -5.375700 1.107500 2.194700

H -3.985700 1.987100 1.517000

O 4.909200 0.186600 -0.632300

C 4.752300 1.053600 -1.544100

H 3.018300 1.329300 -1.874200

H 4.864100 2.125000 -1.319100

H 4.885900 0.779200 -2.601600

**15**

SCF done: -1270.046631

thermal Free Energies -1269.630226

Pd 2.634564 0.246191 -0.793892

P 2.696842 -1.352497 1.110258

C 1.272442 -2.129321 2.025974

H 0.659178 -2.700166 1.324896

H 1.638860 -2.798744 2.812408

H 0.640980 -1.360543 2.479950

C 3.687134 -2.857690 0.637851

H 4.612568 -2.525470 0.161496

H 3.919639 -3.475498 1.512518

H 3.120366 -3.455586 -0.082637

C 3.707505 -0.644113 2.504209

H 3.167824 0.193490 2.956640

H 3.916947 -1.395752 3.273642

H 4.645832 -0.267786 2.089223

C -0.438656 4.202005 -0.033686

C -0.088904 3.104243 -0.820049

C 0.247347 1.863236 -0.247245

C 0.224932 1.777400 1.158315

C -0.131512 2.869191 1.949278

C -0.466612 4.089771 1.357726

H -0.693629 5.145343 -0.510253

H -0.073411 3.204767 -1.903043

H 0.506360 0.844068 1.635057

H -0.142111 2.767751 3.031771

H -0.740131 4.942267 1.973658

C 0.607329 0.710291 -1.144317

H 0.571808 1.071043 -2.172026

C -0.282395 -0.534892 -1.133452

H 0.186524 -1.369634 -1.646358

C -3.966064 -1.416530 -1.115550

C -2.655640 -1.398661 -1.973102

C -1.955785 -2.226389 0.032514

C -3.484324 -1.973139 0.257813

H -4.727925 -2.039803 -1.595697

H -3.985716 -2.901879 0.550035

C -2.003864 -2.696674 -1.441227

H -1.028588 -2.922910 -1.877694

H -2.646225 -3.573678 -1.577017

C -1.762168 -0.298324 -1.393487

H -2.092078 0.733255 -1.486875

C -1.290376 -0.844415 -0.048899

H -1.320357 -0.222915 0.839162

H -2.838380 -1.347297 -3.049063

H -1.528123 -2.926986 0.753924

C -4.578459 -0.059534 -0.798633

C -3.839320 -0.889199 1.266199

O -5.080425 0.729696 -1.574083

O -3.626245 -0.883874 2.463533

N -4.471785 0.148020 0.580869

C -4.905373 1.370774 1.237603

H -5.545242 1.913205 0.540336

H -5.451918 1.120105 2.149680

H -4.042168 1.990219 1.502743

O 4.937234 -0.031233 -0.536429

C 4.858475 0.829179 -1.436797

H 2.701093 1.229638 -2.052847

H 4.910407 1.904421 -1.206249

H 4.938735 0.551658 -2.498686

**17**

SCF done: -1155.531243

thermal Free Energies -1155.143861

Pd 2.154300 -1.490200 -0.519900

P 3.368000 -0.182800 1.249600

C 2.457900 0.560900 2.698300

H 1.879500 -0.216800 3.206100

H 3.151800 1.019500 3.412000

H 1.765500 1.322800 2.330500

C 4.634300 -1.240500 2.128200

H 5.337400 -1.651600 1.397100

H 5.191700 -0.662000 2.873700

H 4.132200 -2.075400 2.626800

C 4.416000 1.250900 0.684700

H 3.774400 2.010000 0.229000

H 4.971900 1.694200 1.519000

H 5.122900 0.900400 -0.073500

C 2.093600 3.019800 -2.127000

C 1.793200 1.670300 -2.294800

C 0.983900 0.967800 -1.375900

C 0.496000 1.693500 -0.268300

C 0.788600 3.048200 -0.102200

C 1.589800 3.721100 -1.027600

H 2.714600 3.528100 -2.860200

H 2.187100 1.137300 -3.156700

H -0.125000 1.200200 0.469700

H 0.382900 3.581700 0.754000

H 1.812700 4.776500 -0.897600

C 0.708300 -0.475400 -1.645500

H 1.004000 -0.689000 -2.673500

C -0.657200 -1.102100 -1.430600

H -0.716800 -2.021500 -2.002800

C -4.259100 -0.473100 -0.475700

C -3.288000 -0.876400 -1.636500

C -2.529400 -2.098900 0.134400

C -3.736500 -1.304100 0.735900

H -5.292600 -0.710300 -0.749900

H -4.483300 -1.995700 1.140600

C -3.105400 -2.371000 -1.276200

H -2.438300 -2.921900 -1.941200

H -4.059500 -2.907900 -1.238200

C -1.921900 -0.260000 -1.310500

H -1.829300 0.821100 -1.343100

C -1.415600 -1.076300 -0.125100

H -0.949400 -0.623900 0.744300

H -3.682400 -0.656000 -2.631200

H -2.241100 -2.972800 0.722800

C -4.207200 0.975900 -0.016000

C -3.407100 -0.271200 1.804500

O -4.519300 1.969400 -0.641800

O -2.944800 -0.456800 2.913800

N -3.729100 0.990100 1.299800

C -3.536800 2.210800 2.063900

H -3.949900 3.033600 1.479000

H -4.047200 2.131000 3.027400

H -2.471900 2.383900 2.249300

H 1.674200 -2.597800 -1.542700

**TS17-4**

SCF done: -1155.531103

thermal Free Energies -1155.146212

Pd 2.258100 0.203800 -0.453700

P 4.255000 -0.613500 0.495200

C 4.312000 -2.311800 1.274900

H 4.042900 -3.065000 0.527800

H 5.307900 -2.541400 1.671200

H 3.583000 -2.364200 2.089500

C 5.699000 -0.709200 -0.680300

H 5.862900 0.275900 -1.126300

H 6.613200 -1.035300 -0.170600

H 5.464200 -1.411800 -1.485300

C 4.959600 0.435600 1.866100

H 4.251400 0.467100 2.699400

H 5.919100 0.042900 2.222300

H 5.098500 1.456600 1.499400

C -1.641800 4.154200 -0.668000

C -0.952000 3.142800 -1.339100

C -0.186900 2.192100 -0.642100

C -0.129400 2.301100 0.757700

C -0.823900 3.305000 1.434300

C -1.585700 4.236400 0.724300

H -2.224000 4.876600 -1.234600

H -1.007600 3.085700 -2.424100

H 0.490900 1.602600 1.314200

H -0.757300 3.367600 2.517700

H -2.120200 5.023300 1.249900

C 0.515200 1.095500 -1.403800

H 0.623600 1.404200 -2.446500

C -0.181300 -0.272300 -1.433800

H 0.310700 -0.952600 -2.123800

C -3.508100 -1.947300 -0.760200

C -2.447800 -1.588000 -1.856400

C -1.175500 -2.353100 -0.125400

C -2.636800 -2.462300 0.425200

H -4.211700 -2.694700 -1.141800

H -2.861100 -3.494700 0.713500

C -1.443900 -2.737600 -1.600000

H -0.560100 -2.720500 -2.241100

H -1.908900 -3.725000 -1.694700

C -1.701800 -0.344800 -1.355500

H -2.250300 0.588900 -1.278200

C -0.850300 -0.857100 -0.202300

H -0.787700 -0.302900 0.728600

H -2.867100 -1.517500 -2.862800

H -0.455700 -2.969800 0.418600

C -4.300400 -0.780000 -0.188000

C -2.978600 -1.544700 1.591500

O -5.112100 -0.079100 -0.759200

O -2.519900 -1.568400 2.717400

N -3.937900 -0.630400 1.153700

C -4.453800 0.436700 1.996500

H -5.446100 0.710400 1.634700

H -4.497600 0.078500 3.026500

H -3.799300 1.314200 1.949600

H 2.181900 1.575900 -1.224000

CH3OH

SCF done: -115.712203

thermal Free Energies -115.683447

H 1.136122 -0.765086 -0.000017

O 0.748273 0.122500 0.000000

C -0.661507 -0.019631 0.000014

H -1.079815 0.990779 -0.002050

H -1.036678 -0.545690 -0.892134

H -1.036773 -0.542221 0.894118

CH2O

SCF done: -114.498216

thermal Free Energies -114.493051

O 0.677528 0.000000 0.000008

C -0.528878 -0.000001 -0.000033

H -1.123477 -0.937975 0.000067

H -1.123478 0.937980 0.000067

Cs2CO3

SCF done: -303.609533

thermal Free Energies -303.631723

O 0.000474 -0.119078 0.029232

C 0.000150 1.217139 0.007549

O -1.124315 1.847488 -0.010375

O 1.124167 1.848375 0.003564

Cs 2.878532 -0.326543 -0.002439

Cs -2.878596 -0.326495 -0.001646

CsCO3-

SCF done: -283.683756

thermal Free Energies -283.702305

O -1.499669 -1.098891 -0.166766

C -2.153494 0.040777 -0.036873

O -3.401213 -0.054630 0.204854

O -1.517542 1.146697 -0.148654

Cs 1.168516 -0.003456 0.020105

CsHCO3

SCF done: -284.318739

thermal Free Energies -284.323915

H -2.265490 1.752374 0.000178

O -1.521246 1.130085 0.002715

C -2.215627 -0.178836 0.000495

O -3.441794 -0.092406 -0.003555

O -1.404424 -1.131615 0.002975

Cs 1.209072 0.001311 -0.000368

Br-

SCF done: -13.237115

thermal Free Energies -13.253291

Br 0.000000 0.000000 0.000000

product

SCF done: -1172.902646

thermal Free Energies -1172.513897

O -1.458470 -0.033721 2.396413

O -2.729141 -2.109384 -1.519476

N -2.328994 -0.833322 0.388693

C -4.000932 0.802779 1.122215

H -3.931663 0.411239 2.129528

C -4.854291 1.866631 0.840896

H -5.454089 2.288078 1.644286

C -4.956457 2.401079 -0.450657

C -4.162376 1.836883 -1.456213

H -4.217731 2.232846 -2.467499

C -3.295351 0.777892 -1.191625

H -2.697080 0.352515 -1.987504

C -3.214266 0.255227 0.102611

C -5.910871 3.533644 -0.749520

H -6.935596 3.164067 -0.889724

H -5.627959 4.065230 -1.664020

H -5.939421 4.260788 0.069692

C -2.156673 -1.946988 -0.462408

C -1.141799 -2.881339 0.175192

H -1.623155 -3.852494 0.331628

C -0.714230 -2.183160 1.493744

H -0.956533 -2.762117 2.391409

C -1.506688 -0.887175 1.535819

C 0.837995 -2.040790 1.345324

H 1.355113 -1.878692 2.293693

C 1.127429 -3.374545 0.614718

H 2.177082 -3.532746 0.359471

H 0.785136 -4.244531 1.185490

C 0.210813 -3.066970 -0.593650

H 0.164828 -3.824038 -1.379896

C 0.669807 -1.673405 -1.037224

H 0.077919 -1.157781 -1.789586

C 1.089562 -0.985126 0.263755

H 0.806785 0.041694 0.480713

C 2.099038 -1.232125 -0.830038

H 2.871120 -1.982355 -0.676627

C 2.599568 -0.030411 -1.628691

H 2.872736 -0.368242 -2.637360

H 1.780694 0.689868 -1.752391

C 3.789261 0.649363 -0.978317

C 3.611696 1.729124 -0.103643

H 2.605729 2.103857 0.078064

C 4.690970 2.347753 0.539175

C 5.981994 1.866949 0.282803

H 6.835970 2.339281 0.763472

C 6.179821 0.795882 -0.588524

H 7.187123 0.437953 -0.786416

C 5.090841 0.189936 -1.216064

H 5.252462 -0.638760 -1.902449

C 4.466834 3.492030 1.501473

H 4.420408 3.135271 2.539277

H 3.525457 4.012005 1.295005

H 5.278352 4.226343 1.448503

PMe3

SCF done: -126.254790

thermal Free Energies -126.170964

P 0.000172 -0.000318 -0.617206

C 1.533341 -0.607403 0.286492

H 2.395119 0.002722 -0.004405

H 1.420100 -0.561926 1.376492

H 1.743845 -1.642324 -0.003623

C -0.240433 1.631371 0.286086

H -1.200721 2.071842 -0.002660

H -0.220717 1.510654 1.376073

H 0.549427 2.331763 -0.005617

C -1.293118 -1.023569 0.286366

H -1.195363 -2.075509 -0.002425

H -1.198817 -0.946013 1.376363

H -2.294196 -0.688834 -0.005763

substrate(Br)

SCF done: -322.209778

thermal Free Energies -322.120371

C 3.511743 0.485801 0.000290

C 2.332191 1.225241 0.000136

C 1.069996 0.597023 -0.000154

C 1.036245 -0.811441 -0.000356

C 2.217982 -1.549645 -0.000210

C 3.459458 -0.909386 0.000134

H 4.470036 0.998144 0.000529

H 2.381417 2.311752 0.000242

H 0.086493 -1.329708 -0.000667

H 2.166781 -2.635217 -0.000371

H 4.376457 -1.492348 0.000253

C -0.106927 1.473118 -0.000253

H 0.148798 2.534203 -0.000377

C -1.432634 1.274373 -0.000196

H -2.119269 2.111285 -0.000247

Br -2.401116 -0.377389 0.000123

alkene

SCF done: -592.873353

thermal Free Energies -592.711147

C 0.430779 0.774789 -0.914962

C 1.683873 1.131636 -0.025289

C 1.682917 -1.132660 -0.024957

C 0.430454 -0.774984 -0.914861

H 0.537881 1.215769 -1.910981

H 0.537352 -1.215794 -1.911002

C 2.639293 -0.000983 -0.480238

H 3.585628 -0.001126 0.068933

H 2.837920 -0.001405 -1.559129

C 1.379657 0.670437 1.393787

H 1.110419 1.330814 2.211342

C 1.379690 -0.670673 1.394200

H 1.110404 -1.330444 2.212189

H 2.022185 2.162300 -0.140526

H 2.020112 -2.163730 -0.139818

C -0.919472 1.172689 -0.337336

C -0.920393 -1.172100 -0.337408

O -1.345090 2.293480 -0.139670

O -1.347440 -2.292291 -0.139885

N -1.626691 0.000493 -0.058125

C -2.952107 0.000145 0.538812

H -3.489274 -0.884983 0.193649

H -2.886442 -0.027414 1.632010

H -3.467254 0.913280 0.236149

CsCO3Cs2CO3

SCF done: -587.356685

thermal Free Energies -587.381109

O 4.413458 1.861708 0.393031

C 3.199325 1.363169 0.386528

O 2.230025 2.093218 0.792295

O 3.017204 0.137250 -0.029801

Cs 0.002196 0.048177 0.511437

C -3.172528 -1.324218 0.454349

O -4.371699 -1.857386 0.464589

O -3.018506 -0.113300 -0.013985

O -2.190370 -2.007527 0.907492

Cs -5.814984 0.373614 -0.492598

Cs 5.798211 -0.442617 -0.476189
